# Association mapping identifies quantitative trait loci (QTL) for digestibility in rice straw

**DOI:** 10.1186/s13068-020-01807-8

**Published:** 2020-10-08

**Authors:** Duong T. Nguyen, Leonardo D. Gomez, Andrea Harper, Claire Halpin, Robbie Waugh, Rachael Simister, Caragh Whitehead, Helena Oakey, Huong T. Nguyen, Tuat V. Nguyen, Tu X. Duong, Simon J. McQueen-Mason

**Affiliations:** 1 Plant Biotechnology Division,, Field Crops Research Institute (FCRI), Hai Duong, Vietnam; 2 Centre for Novel Agricultural Products (CNAP), University of York (UoY), Wentworth Way, York, UK; 3 Division of Plant Sciences, School of Life Sciences, University of Dundee (UoD), Dundee, UK; 4 Cell, and Molecular Genetics, The James Hutton Institute (JHI), Invergowrie Dundee, UK; 5 School of Agriculture Food and Wine, University of Adelaide, Waite Campus, Adelaide, SA Australia; 6 Vietnam Academy of Agricultural Sciences, Hanoi, Vietnam; 7 School of Agriculture and Environment, University of Western Australia (UWA), Crawley, WA Australia

**Keywords:** Rice (*oryza sativa*), Lignocellulose, Biomass, Saccharification, Digestibility, GWAS, QTL

## Abstract

**Background:**

The conversion of lignocellulosic biomass from agricultural waste into biofuels and chemicals is considered a promising way to provide sustainable low carbon products without compromising food security. However, the use of lignocellulosic biomass for biofuel and chemical production is limited by the cost-effectiveness of the production process due to its recalcitrance to enzymatic hydrolysis and fermentable sugar release (i.e., saccharification). Rice straw is a particularly attractive feedstock because millions of tons are currently burned in the field each year for disposal. The aim of this study was to explore the underlying natural genetic variation that impacts the recalcitrance of rice (*Oryza sativa*) straw to enzymatic saccharification. Ultimately, we wanted to investigate whether we could identify genetic markers that could be used in rice breeding to improve commercial cultivars for this trait. Here, we describe the development and characterization of a Vietnamese rice genome-wide association panel, high-throughput analysis of rice straw saccharification and lignin content, and the results from preliminary genome-wide association studies (GWAS) of the combined data sets. We identify both QTL and plausible candidate genes that may have an impact on the saccharification of rice straw.

**Results:**

We assembled a diversity panel comprising 151 rice genotypes (*Indica* and *Japonica* types) from commercial, historical elite cultivars, and traditional landraces grown in Vietnam. The diversity panel was genotyped using genotype by sequencing (GBS) methods yielding a total of 328,915 single nucleotide polymorphisms (SNPs). We collected phenotypic data from stems of these 151 genotypes for biomass saccharification and lignin content. Using GWAS on the *indica* genotypes over 2 years we identified ten significant QTL for saccharification (digestibility) and seven significant QTL for lignin. One QTL on chromosome 11 occurred in both GWAS for digestibility and for lignin. Seven QTL for digestibility, on CH2, CH6, CH7, CH8, and CH11, were observed in both years of the study. The QTL regions for saccharification include three potential candidate genes that have been previously reported to influence digestibility: *OsAT10*; *OsIRX9*; and *OsMYB58/63-L*.

**Conclusions:**

Despite the difficulties associated with multi-phasic analysis of complex traits in novel germplasm, a moderate resolution GWAS successfully identified genetic associations encompassing both known and/or novel genes involved in determining the saccharification potential and lignin content of rice straw. Plausible candidates within QTL regions, in particular those with roles in cell wall biosynthesis, were identified but will require validation to confirm their value for application in rice breeding.

## Background

The need to cut carbon emissions has become a global priority and the production of low carbon liquid fuels and chemicals are important components in the drive for a sustainable industrial bio-economy. The use of major crops and agricultural land exclusively for biofuel production is considered unsustainable and generates concerns over global food security. However, the use of non-food crop residues represents an alternative source of biomass. Such lignocellulosic crop biomass is typically composed of around 70% polysaccharides that can be potentially depolymerized to produce sugars for fermentation. Millions of tons of rice straw are burned every year for disposal [1]. Field burning of biomass generates ground-level atmospheric pollution that is responsible for premature mortalities, lost economic activity and decreased agricultural yields in many rice-growing nations [2]. Consequently, there are clear benefits to valorizing rice straw and other residues to produce fuels and chemicals. However, the use of biomass is hindered by its recalcitrance to digestion.

Most agriculturally important broad-acre cereals have large complex genomes that make them complicated to use for research purposes. One exception to this is rice (*Oryza sativa*), one of the worlds’ most important cereal crops. Rice has a small diploid genome (only about twice the size of *Arabidopsis*) and well-developed molecular genetic tools [3].

Albeit with many advantages, most research focused on understanding the synthesis and construction of plant cell walls has been conducted in *Arabidopsis* [4]. Unfortunately, many aspects of this research cannot be directly transferred to grasses, as monocots and dicots differ in their cell wall biology [5]. While they both comprise cellulose microfibrils embedded in a matrix of hemicellulose and lignin, there are substantial differences in these two components and how they bond to one another. While the predominant hemicellulose in dicot lignocellulose is an acetylated glucuronoxylan, grasses have more complex, highly decorated arabinoxylans [6]. Grass arabinoxylans are notably decorated with hydroxycinnamic acid esters associated with arabinosyl side chains. Ferulic acid esters on arabinoxylans form cross links with neighbouring stretches of different arabinoxylan chains and with lignin [7], a feature not found in dicots. Lignin structure also differs considerably between dicots and grasses, with a greater preponderance of hydroxycinnamic acids in grass lignin [8].

Alterations in cell wall components can affect the recalcitrance of lignocellulosic biomass, and thus improve its saccharification with the potential to improve energy crops through plant breeding [9–11]. While reducing lignin can decrease recalcitrance in grasses [12], several publications also indicate that alterations in hydroxycinnamic esters can have a significant effect on recalcitrance [13]. In rice and *Brachypodium*, decreased levels of ferulic acid accompany increases in lignocellulose digestibility [14–16]

Recently, important advances that lay the foundations for engineering or breeding plants for biofuel production have been made. These include lists of genes that could be manipulated or mined towards a goal of pathway engineering. However, for practical implementation, many challenges remain to be addressed [17]. In plants and animals, studies of genetic sources of phenotypic variation have been the key to determining the cause of disease, improving agriculture and understanding adaptive processes [18]. In particular, genetic analysis of natural variation has been used to identify both genes and quantitative trait loci (QTL) that account for significant amounts of phenotypic variation for a given trait within a population. QTL were originally mapped in bi-parental populations in plants [19]. In bi-parental mapping populations, genetic resolution is often limited, confined to a range of 10 cM to 30 cM due to the restricted number of meiotic events captured during a cross between two parental lines [20]. For example, Truntzler et al. identified 26 and 42 QTL in a maize bi-parental population that accounted for much of the variation in forage digestibility and cell wall composition traits, respectively, apparent in that population [21]. Penning et al. similarly identified QTL for cellulase digestibility in a recombinant inbred population of maize [22], and Liu et al. identified a broad region on chromosome 1 that influenced digestibility in rice straw in a bi-parental population [23]. Unfortunately, the number, effect and resolution of individual QTL in a bi-parental population frequently hamper causal gene identification. In addition, only a couple of all possible alleles present in a species can be examined for linkage to a trait in a population derived from two parental individuals [24].

Linkage disequilibrium (LD) mapping, or association mapping (AM) exploits historical recombination events that have occurred in all of the genomes contained within a population. All major alleles segregating in those genomes can then be considered when attempting to identify significant marker–phenotype associations [25]. Over the last few years, genome-wide association studies (GWAS) have become increasingly popular. GWAS is a powerful approach that overcomes many of the constraints inherent to bi-parent linkage mapping. It exploits the considerable variation revealed by high-throughput molecular markers in natural or constructed populations across all chromosomes with high resolution [26]. An appropriate panel of genotypes, density of molecular markers and high-quality phenotypic data are key to establishing successful association study. GWAS was first applied in humans [27] and, after over two decades, is continuing to provide a powerful approach for the localization of genes underlying both simple and complex traits in many species, including crops. The advent of high-density single-nucleotide polymorphism (SNP) genotyping is allowing whole-genome scans to identify small haplotype blocks that are significantly correlated with quantitative trait variation [18]. GWAS in crops usually use a population of diverse (and preferably homozygous) genotypes that is genotyped once and can be phenotyped for many traits to generate specific mapping populations for specific traits or QTL [28]. There have been a number of studies using a range of genetic approaches to identify QTL for digestibility with different degrees of resolution in different species such as sorghum [29], *Miscanthus* [30], maize [22], alfalfa [31], and poplar [32]. Nevertheless, digestibility/saccharification is a difficult trait to measure, with potential variation arising from both the field and the laboratory phases of the work [33].

Rice is a selfing species and, like *Arabidopsis*, a good candidate for GWAS. Huang et al. identified an unbiased set of common SNPs that was used to identify strong associations between genetic loci and 14 agronomic traits, including heading date, grain size, and starch quality [34]. With the now well-developed molecular genetics tools, the advent of affordable large-scale DNA sequencing and association genetic studies starting to reach their full potential, GWAS in rice has the potential to identify both QTL for saccharification and novel genes involved in cell wall synthesis.

The aim of the present work was to determine whether GWAS can be used to identify QTL and candidate genes associated with the saccharification potential of rice straw. Using a new association panel comprising 151 rice genotypes from Vietnam, we measure lignocellulose digestibility and lignin content in field-grown straw from this population across 2 years. Association studies using only the *indica* subset revealed a number of significant QTL and candidate genes, some common to both lignin content and digestibility.

## Results

### SNP identification

**Fig. 1:**
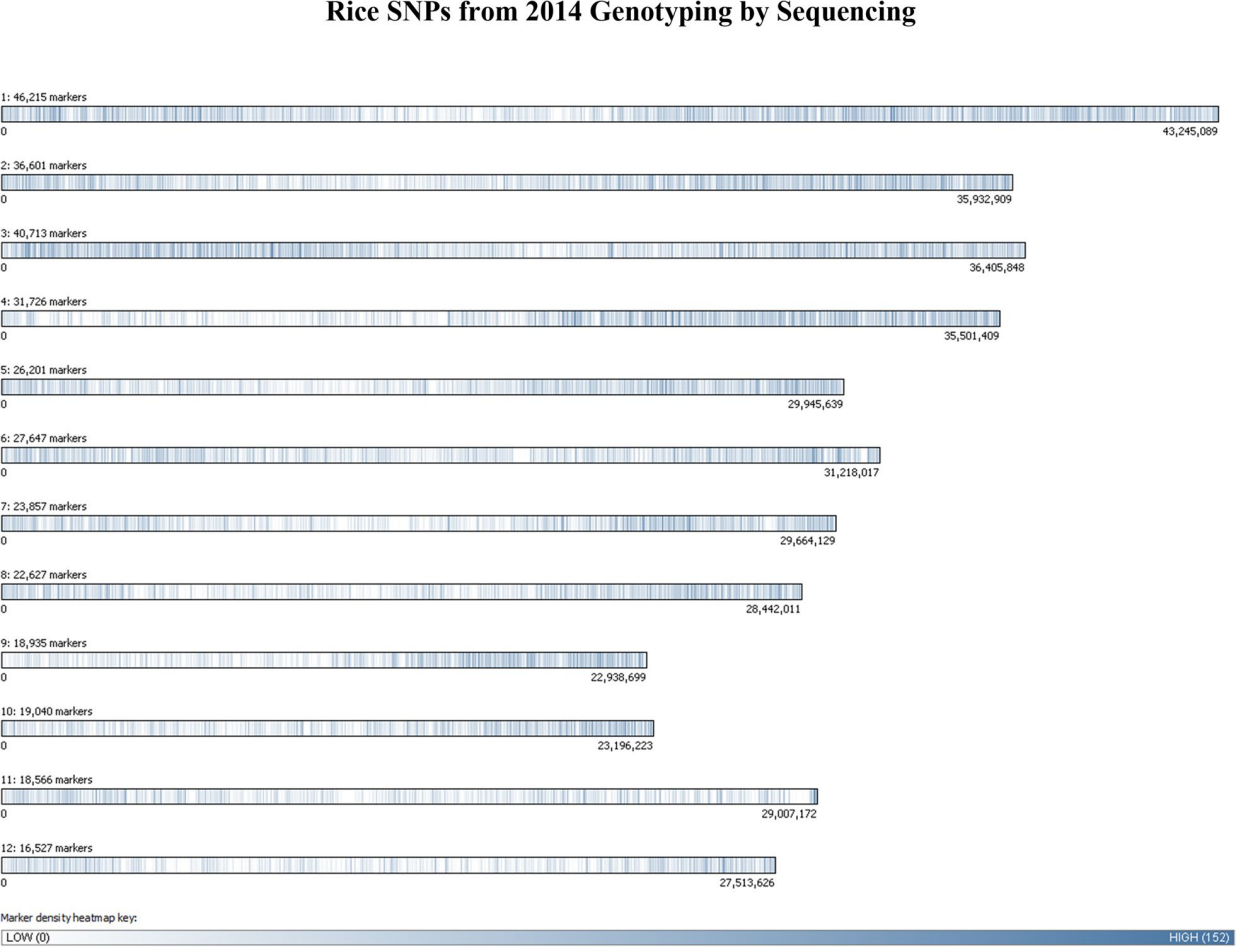
Bar graph showing the distribution of identified SNPs across the rice genome

The SNP matrix used for association mapping in the present work was generated by genotyping by sequencing (GBS) 172 rice genotypes, followed by GBS “Discovery Pipeline” analysis (Tassel Version: 3.0.166, date: April 17, 2014). We identified a total of 328,915 SNPs that were stored in HapMap [35] and used as genotypic data for GWAS (Fig. 1). The average density of SNP markers in our panel is 1SNP/Kb. It has been reported that genome-wide linkage disequilibrium decay rates for rice subspecies such as *indica* and *japonica* are estimated at ~ 123 kb and ~ 167 kb [34], and cultivated rice has a longer range of decay (100 kb to over 200 kb) [36]. For GWAS studies, the coverage of markers that we generated should therefore give satisfactory resolution. Indeed, this SNP density means that causative polymorphisms stand a reasonable chance of being in LD with one or more markers and should help to identify small haplotype blocks that are significantly correlated with complex traits such as lignocellulose recalcitrance.

### Population stratification

**Fig. 2:**
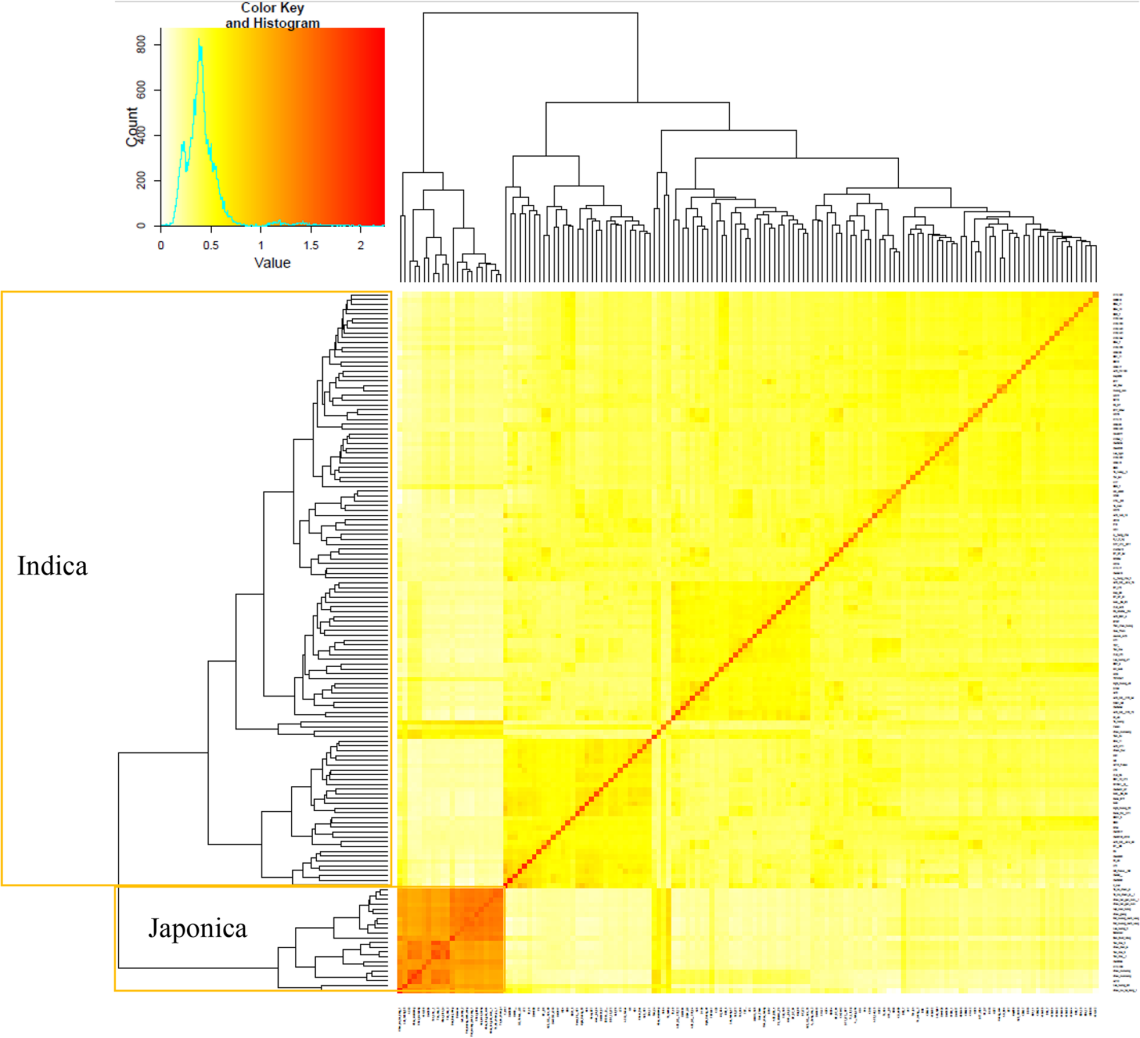
Phylogenetic tree in the form of a kinship plot. A heat map of the values in the kinship matrix, showing the level of relatedness among the population (the darker area showing highly related variety and also from different origin with the rest of the population). The population is separated into the main population (*Indica)* in the bigger orange box, and subpopulation (*Japonica*) in the smaller orange box

From 172 genotypes used for SNP identification, we reduced the number for GWAS to 151 due to appearance of some identical genotypes. Controlling for population structure is a standard procedure in GWAS and is particularly important in this research as genotypes were collected from many different sources and include both *indica* and *tropical japonica* varieties. The diversity level and stratification of the population were examined before performing GWAS. A phylogenetic tree and heat map of the values in the kinship matrix created from the SNPs, which both show relatedness among the population were calculated using GAPIT (Fig. 2) [37, 38]. The results show that there are two subpopulations in the association mapping panel (Fig. 2). The smaller subpopulation includes 22 *tropical japonica* genotypes with the other subpopulation comprising 129 *indica* genotypes.

### Measuring lignocellulose recalcitrance and lignin content

#### Recalcitrance

**Fig. 3:**
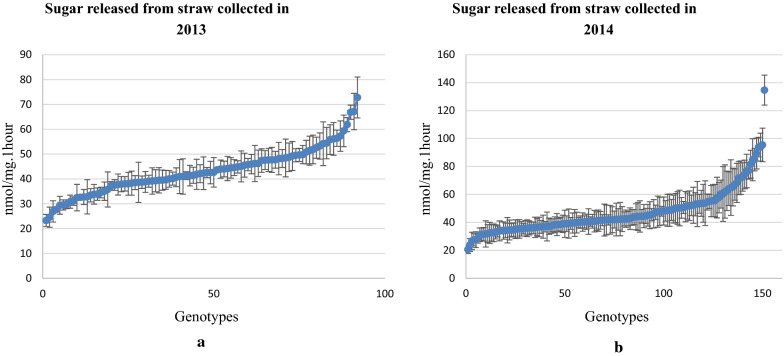
Range of saccharification values obtained for the rice association panels in 2013 (**a**) and 2014 (**b**). Error bars represent the STDEV of each genotype

**Fig. 4:**
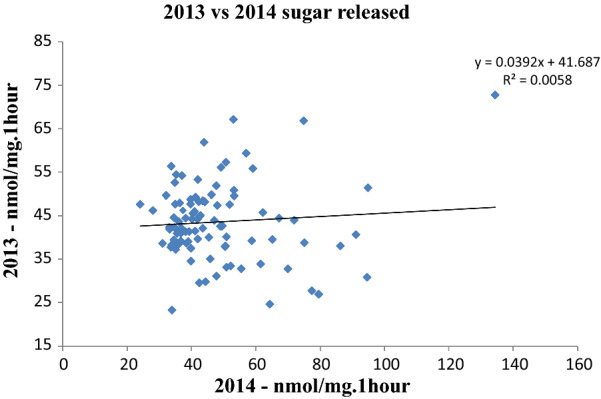
Correlation between the results for saccharification between trials in 2013 and 2014 for 93 varieties present in both years

Lignocellulose recalcitrance to digestion was measured by incubating ground straw from individual genotypes with a commercial cellulase cocktail following a water pre-treatment at 94 °C using an automated platform [39]. To determine QTL for recalcitrance in our rice association panel, we harvested straw over two consecutive years during the spring season in 2013 (93 genotypes) and the summer season in 2014 (151 genotypes). The results from the 2014 harvest showed values in the range of 20–134 nmol of reducing sugar equivalents/mg of biomass per hour of hydrolysis (nmol/mg h), and for the 2013 harvest the range was between 23 and 72.8 nmol/mg h (Fig. 3). There is little correlation between the saccharification data sets from both years in the 93 genotypes present in both trials (Fig. 4). We attribute the lack of correlation between two datasets largely to environmental effects of growth in different seasons on saccharification. This illustrates the difficulties inherent in measuring complex traits where field and laboratory phases of the analysis and different years of growth can introduce non-genetic variation. In addition to that, there is also potential influence of different environmental conditions to marker effects (i.e. marker by environment interaction effects) [33] Most rice genotypes are adapted for optimal growth in a specific growing season, while some are adapted for both seasons, causing differences in biomass quality.

#### Lignin content

**Fig. 5:**
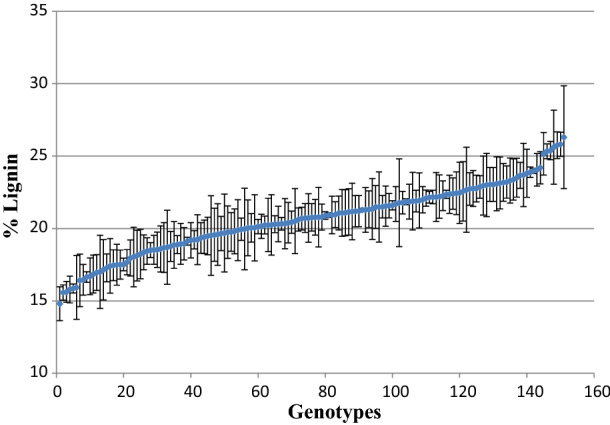
Total lignin content across the rice association panel. Lignin was measured using the acetyl bromide method in 151 genotypes, with three biological replicates per genotype. Error bars represent the STDEV for each genotype

Lignin content was assessed using the acetyl bromide method [40] and showed a significant degree of variation among the 151 rice genotypes included in the association panel, ranging between 26.3% and 14.3% (Fig. 5).

**Fig. 6:**
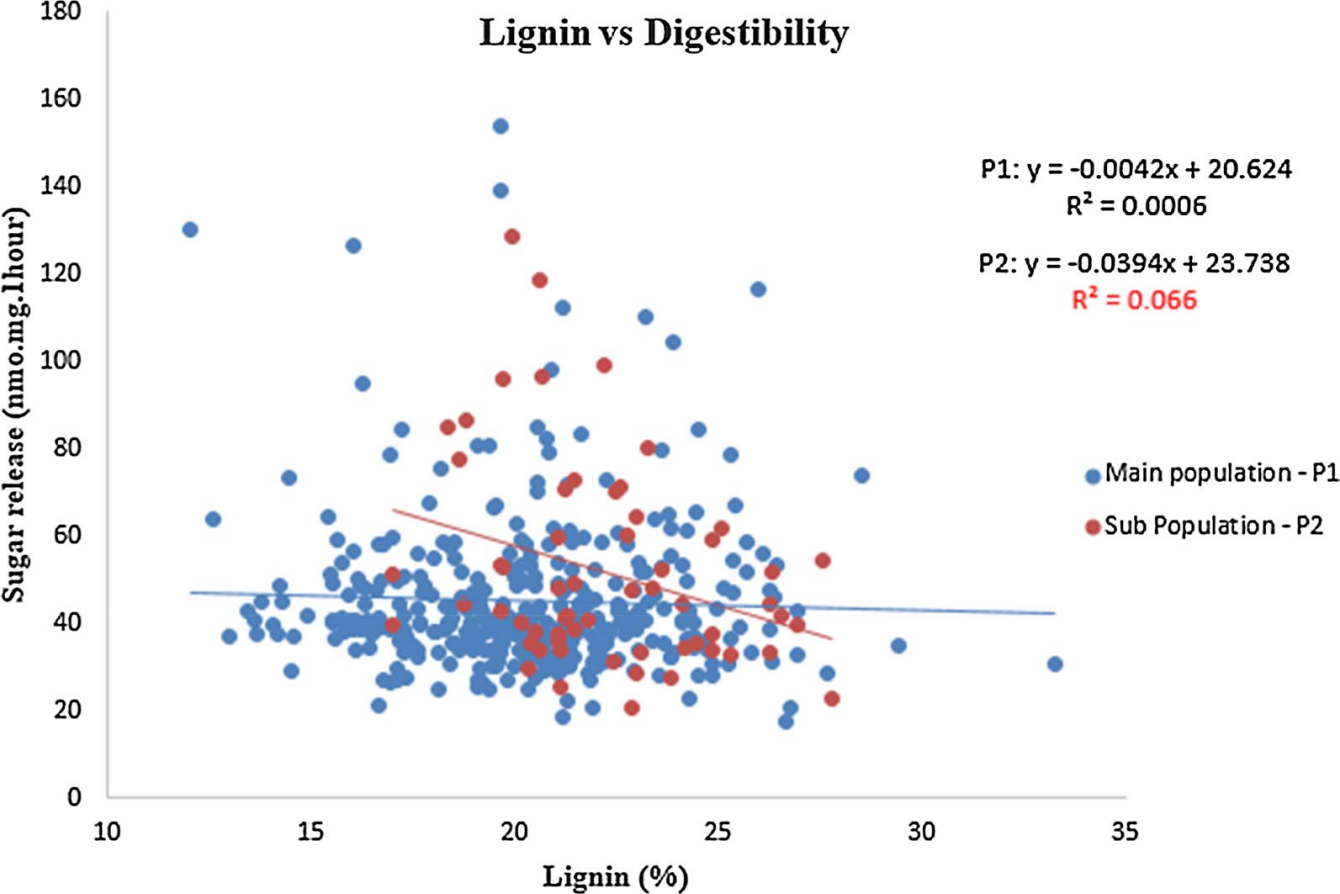
Correlation graph of digestibility vs lignin observing 151 genotypes, three biological reps. Blue dots represent the main population P1 (*Indica* rice genotypes) and red dots represent subpopulation P2 (*Janopica* rice genotypes)

A correlation analysis between lignin content and recalcitrance revealed no significant correlation between the two for the *indica* population (*R*^2^ = 0.0006), although there was a significant correlation apparent in the smaller *japonica* sub-population (*R*^2^ = 0.066, and the *p* = 0.045*) (Fig. 6). Based on these results, we decided to remove the *japonica* subpopulation to improve the power of GWAS and to avoid the population structure misleading the analysis [18].

### GWAS for recalcitrance

**Fig. 7:**
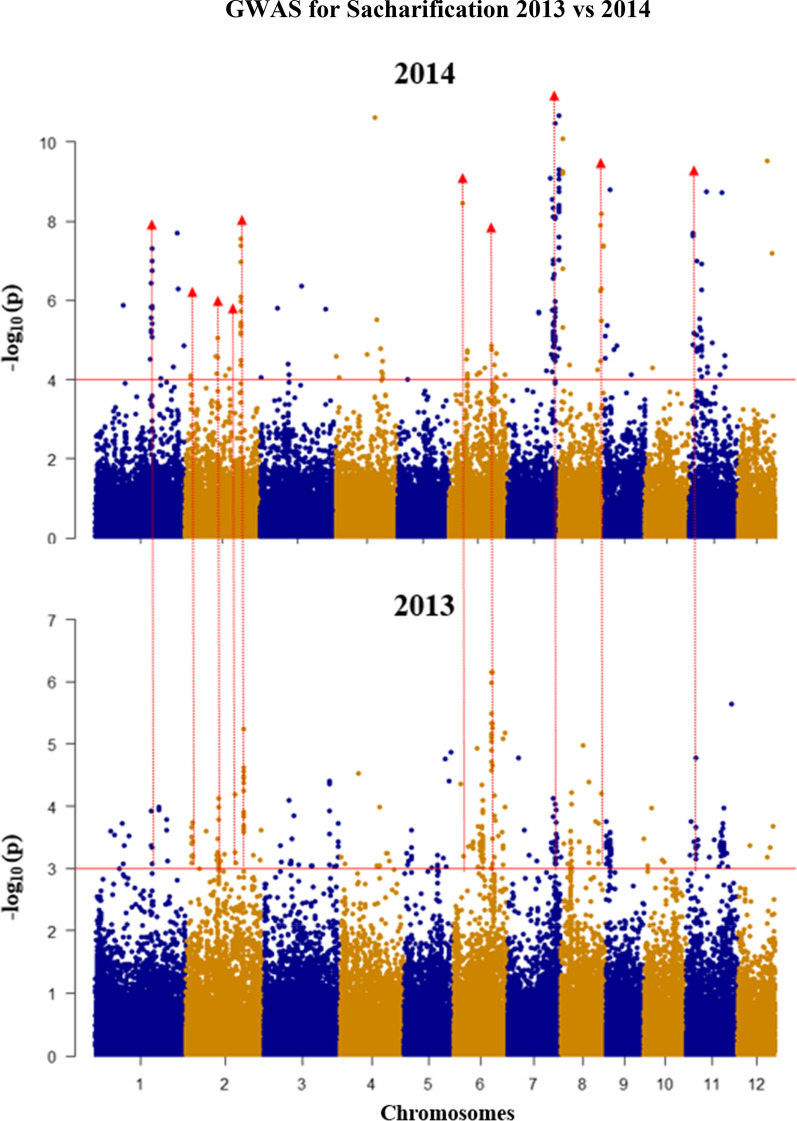
Genome-wide association study shows association between saccharification and markers across rice genome over 2 years of studies. Manhattan plot shows significant SNPs for saccharification (significant SNPs with *p* < 0.001; MAF > 5%); the red arrow indicates the common QTL. Red line indicates cutoff for significant SNP with a false discovery rate (FDR) of < 0.05

We ran GWAS for recalcitrance in 2 years separately, using adjusted saccharification genotype means from straw biomass harvested from 83 *indica* genotypes in 2013 and 125 *indica* genotypes in 2014. A separate mixed linear model (MLM) was fitted for each year separately in TASSEL [41]. We identified several significant associations in each year including seven QTL regions, on CH2, CH6, CH7, CH8, and CH11, present in both years’ data (Table 1). The data set from 2014 yielded a total of 102 significant SNP associations (Table 1). Figure 7 shows a Manhattan plot showing QTL for saccharification with a false discovery rate (FDR) of < 0.05, as the cutoff for significant SNPs (above the red line). The quantile–quantile (QQ) plot that represents deviation of the observed P values from the null hypothesis is shown in Additional file 1. The genetic effects of these QTL to phenotype variance were calculated as phenotypic variance explained (PVE) by significant SNPs (see Table 1). There are SNP clusters/QTL on CH1, CH2, CH6, CH7, CH8, and CH11, which have PVE values ranging from 18% (at CH2_24.6 ± 0.2 Mb) to 56% (at CH7_26.4 ± 0.4 Mb) (Table 1).

**Table 1:** Digestibility QTL regions, the significant SNPs, and selected candidate genes in the QTL regions in 2014; the significant SNPs are selected by false discovery rate (FDR) < 0.05

Chromosome (CH)	QTL regions (Mbp)	No of significant SNPs in QTL regions	Most significant *p* value of SNP in QTL regions	MAF^a^	R2 (%)^b^	Candidate genes
1	CH1_29.5 ± 0.2	9	4.92E−08	0.179	22.6	LOC_Os01g51260 (*OsMYB26 TF*) [79] LOC_Os01g50720 Homologous to *BdMYB48* [80, 81]
2	CH2_2.9 ± 0.2	2	8.04E−05	0.18	13.8	
	CH2_19.2 ± 0.2	2	8.94E−06	0.2	17.3	
	CH2_24.4 ± 0.2	1	5.30E−05	0.13	13.1	
	CH2_28.5 ± 0.2	14	4.06E−08	0.191	25.9	LOC_Os02g46970 (*4CL2*) [82]; LOC_Os02g46780 (*OsMYB58/63 L*) [46]
6	CH6_6.2 ± 0.2	1	3.61E−09	0.23	30.0	
	CH6_23.4 ± 0.2	2	1.37E−05	0.23	15.7	LOC_Os06g39470 (BADH)
						LOC_Os06g39390 *(OsAT10)* [16, 43]; LOC_Os06g39970 *(CESA11)* [83]
7	CH7_26.2 ± 0.2	9	2.78E−09	0.18	27.5	
	CH7_27.5 ± 0.2	12	3.34E−11	0.14	39.1	
	CH7_29.4 ± 0.2	8	2.17E−11	0.14	36.0	Os07g49370 *(OsIRX9) * [44]*:*
8	CH8_2.1 ± 0.2	5	8.34E−11	0.18	30.1	
	CH8_26.8 ± 0.2	5	1.30E−08	0.09	26.8	
	CH8_27.3 ± 0.2	3	6.38E−09	0.2	28.3	LOC_Os08g43040 and LOC_Os08g43020(Orthologous to AT5G48930, *HCT*)
	CH8_28.0 ± 0.2	5	4.15E−08	0.17	22.5	
11	CH11_2.3 ± 0.2	6	2.05E−08	0.16	24.1	
	CH11_4.1 ± 0.2	8	7.51E−06	0.2	15.5	LOC_Os11g07960 (Orthologous to AT5G48930, *HCT*)
	CH11_5.1 ± 0.2	1	3.00E−06	0.18	18.1	
	CH11_6.3 ± 0.2	9	5.56E−07	0.17	19.01	

^a^Minimum allele frequency

^b^Phenotypic variance explained (PVE) by significant SNP

### GWAS for lignin content

**Fig. 8:**
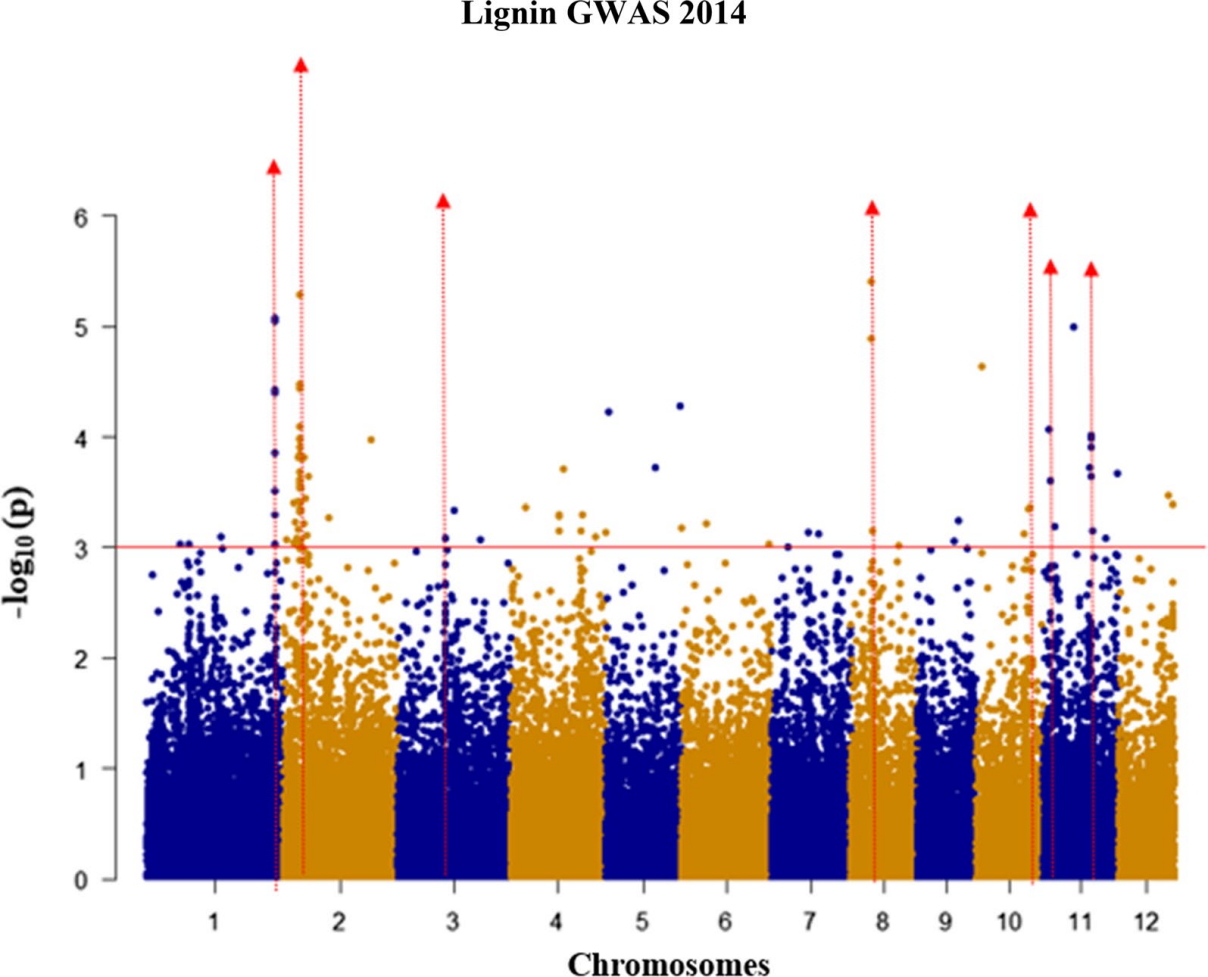
Genome-wide association study showing association between lignin content and SNP markers across the rice genome. Manhattan plot showing lignin QTL Significant SNP (*p* < 0.001; MAF > 5%). Red line indicates cutoff for significant SNP at *p* < 0.001

By fitting the adjusted means of lignin of 124 *indica* genotypes grown in 2014 in the same GWAS model as for recalcitrance, we found 56 significant SNPs using a cutoff at *p* < 0.001 and MAF > 0.05. The FDR correction for *p* value was not applied because none of the SNPs qualified for FDR < 0.05. In this case, we used only the *p* value to account for the significance of each SNP associated with lignin content. This means that we have accepted an overestimate of the true significance of some SNPs and accept that some may be false positives. The QQ plot that represents deviation of the observed *p* values from the null hypothesis is shown in Additional file 1. The significantly associated SNPs with lignin content are situated in CH1, CH2, CH3, CH8, CH10, and CH11 (Table 2). These significant SNPs explain from 5.18% (at CH10_19.2 ± 0.3 Mb) to 12.58% (at CH11_4.0 ± 0.2 Mb) of the phenotypic variation (Table 2). The QTL on CH11_4.0 ± 0.2 Mb is at the same region as a QTL found in GWAS for digestibility, although no common significant SNPs were found between these two GWAS (Fig. 8, Tables 1 and 2).

**Table 2:** Lignin QTL regions, the significant SNPs, and candidates in the QTL regions in 2014; the significant SNPs are selected by *p* value < 0.001 equal to Log10*p* value > 3.0

Chromosome (CH)	QTL regions (Mbp)	No of significant SNPs in QTL regions	Most significant *p* value of SNP in QTL regions	MAF^a^	R2 (%)^b^	Candidate genes
1	CH1_41.0 ± 0.2	9	8.96E−06	0.24	15.9	
2	CH2_5.5 ± 0.2	24	5.19E−06	0.47	17.4	
3	CH3_14.7 ± 0.2	1	8.21E−04	0.3	9.18	7 peroxidases
8	CH8_8.8 ± 0.2	3	3.98E−06	0.38	16.2	
10	CH10_19.4 ± 0.2	2	4.48E−04	0.44	9.5	
	CH11_4.0 ± 0.2	2	6.44E−04	0.17	12.58	*HCT*
11	CH11_18.8 ± 0.2	8	9.69E−05	0.38	12.6	LOC_Os11g47390.1 putative laccase 14

^a^Minimum allele frequency

^b^Phenotypic variance explained (PVE) by significant SNP

### Identification of candidate genes

#### Candidate genes for recalcitrance

To identify the candidate genes underlying the QTL, we searched within 400 kb (± 200 kb of the peak SNPs) around the significant loci identified, based on the linkage disequilibrium (LD) decay range, published for rice [36, 42]. The MSU Rice Genome Annotation Project (https://rice.plantbiology.msu.edu/expression.shtml) database was used to search for genes and their expression data in these regions (Additional file 2). Candidates were selected based on whether the function of the genes had been characterized before in rice or if similar genes in other species had known roles in cell wall biosynthesis or modification. Table 2 shows the candidates identified for each saccharification QTL. Three candidate genes located in QTL regions found in both years of harvest have previously been shown to affect lignocellulose digestibility. The first one, LOC_Os06g39390 (*OsAT10*) encoding a *p*-coumaroyl coenzyme A transferase belongs to the Mitchell clade of BADH acyl transferases and has previously been shown to add *p*-coumaroyl esters to arabinoxylan [16]. This gene and its close neighbour, locus *LOC_Os06g39470* (OsAT8), belong to family PF02458 transferases [10, 43]. In 2010, Piston et al. showed that cell walls of lines where both genes are down-regulated exhibit a reduced content of ester-linked ferulate [43]. A candidate gene located within the QTL region on chromosome 7 is LOC_Os07g49370 (*OsIRX9*) that encodes a glycosyl transferase involved in the synthesis of the xylan backbone in the secondary and primary cell walls. Expressing *OsIRX9* in an Arabidopsis *irx9* mutant background restored xylosyltransferase activity and stem strength to wild-type levels [44]. A candidate gene within the QTL on chromosome 2 is locus LOC_Os02g46780 next to the SNP-S2_28582605 (*p* = 1.05E−07), identified as *OsMYB58/63 L* [45], which is a homologous to the Myb transcription factor *OsMYB58/63* involved in the expression of a rice secondary wall-specific cellulose synthase gene, *OsCesA7* [46].

Table 1 lists the QTL regions along with the positions of the three candidates mentioned above, and a number of other potential candidate genes.

#### Candidate genes for lignin content

All genes located in QTL regions and their expression data are listed in Additional file 3. Candidate genes associated with lignin content QTL were identified following the same procedure as for recalcitrance. The list of candidate genes in the QTL regions is shown in Table 2. Several QTL regions encompass genes known to be involved in lignin biosynthesis. A *hydroxycinnamoyltransferase* (*HCT*) gene on CH11 (CH11_4.0 ± 0.2 Mb) is in the common QTL region between GWAS for recalcitrance and lignin content. Interestingly, there are also two potential HCT genes located within a digestibility QTL on chromosome 8, namely, LOC_Os08g43040 and LOC_Os08g43020 (Table 2). Reduced expression of *HCT* in alfalfa has been shown to increase stem digestibility [47].

There is a cluster of seven peroxidase genes located close to the peak in the lignin QTL region on CH3_14.5 ± 0.4. Also, a laccase, LOC_Os11g47390.1, located in the QTL region CH11_18.8 ± 0.3, is surrounded by several cell wall genes, including a wall-associated kinase (*WAK*), a kinase, a receptor-like protein kinase, and a glycosyl hydrolase. Peroxidases together with laccases have been proposed to take part in the polymerization of monolignols into lignin [48]. Downregulation or disruption of these enzymes led to the reduction of lignin content in plants [48–50].

## Discussion

The lignin content in our rice accession straws are at a similar level to that of grasses in general and higher than in dicot but lower than in wood species [5, 49–51]. A comparison of our results with the other unpublished data (using the same method) in our laboratory shows that rice has a top high lignin content and has the highest range of digestibility in the studied grasses.

We have piloted the use of GWAS to identify QTL for the saccharification potential of rice straw using an association panel of 151 Vietnamese elite and landrace genotypes. In this association panel, based on the pairwise studies for relatedness among all the genotypes, 129 *indica* genotypes were grouped into the main population and 22 tropical *japonica* genotypes were grouped into a smaller group, which can be considered as a sub-population. The *japonica* sub-population was removed from all GWAS to reduce the number of confounding factors. False positives and negatives in GWAS can occur when the patterns of population structure overlap with patterns of the phenotype and with patterns in environmental variation [18].

We used an automated multi-phasic saccharification platform to phenotype the straw samples collected over two different growing seasons (spring and summer) in 2 years (2013 and 2014), [52]. Only eight genotypes in the top of 25% for digestibility in 2013 were found in the top 25% in 2014. We attribute this to the environmental effects on the population including variation in day length requirement for different genotypes [53, 54]. Despite this apparent lack of correlation, we nevertheless identified seven QTL that were common across both years. There have been a number of studies using different genetic approaches to identify QTL for saccharification in different types of plant biomass. Only a few candidate genes have been identified and validated from association mapping for saccharification so far. In alfalfa, 20 simple sequence repeat (SSR) markers were predicted to be associated with fiber-related quality traits (heritability, *H*^2^ = 45 to 73.6); no specific candidate genes were reported but their finding helped to facilitate marker-assisted breeding programs [31]. In sorghum, screening 703 SSR markers against low and high saccharification (glucose release by cellulase) pools identified two markers on the sorghum chromosomes 2 (23–1062) and 4 (74-508c) associated with saccharification yield; these markers were physically close to genes encoding plant cell wall synthesis enzymes such as xyloglucan fucosyltransferase (149 kb from 74-508c) and UDP-d-glucose 4-epimerase (46 kb from 23-1062) [29]. In maize, recombinant inbred lines screened for lignin abundance and sugar yield established 11 QTL, using pyrolysis molecular-beam mass spectrometry to establish stem lignin content and an enzymatic hydrolysis assay to measure glucose and xylose yield [22]. So far, several naturally occurring mutants with reduced lignin have been identified in cereals such as *brown midrib (bm*) mutants in maize [55], *orange lemma *(*rob*) mutants in barley [56], and “*gold hull internode*” (*gh*) mutant in rice [57]. The phenotypes with reduction and changes in lignin characteristic of these mutants has shown their potential impacts on cell wall digestibility [58–61]. In the present work, we have used a direct GWAS approach in an association panel to screen for QTL in rice and found a number of genes already established as affecting saccharification, as well as other novel candidates.

By screening the regions in close proximity to the significant SNPs in the seven 2-year QTL, as well as two single-year QTL, we identified 12 candidate genes, which included the transcription factors, *OsMYB26 TF, OsMYB58/63 L*, and an ortholog of *BdMYB48.* The other candidate genes are *OsHCT2,* three homologs of *HCT, Os4CL2, OsCESA11, OsAT8, OsAT10* (BAHD family), and *OsIRX9 *(a GT43)*. OsAT10, OsIRX9*, and *OsMYB58/63L* were detected in both years of assays.

Association mapping based on examining individual genes and alleles at the loci responsible for lignin content has been applied to perennial ryegrass to identify significantly associated SNPs. An intronic SNP in the candidate gene *LpCCR1* in poplar was found significantly associated with cell wall digestibility and Klason lignin content in stem material [62]. Similarly, association mapping across 40 candidate genes associated with lignin content were characterized by pyrolysis molecular-beam mass spectrometry (PyMBMS), and 13 significant single marker associations were found for 9 candidate genes in black cottonwood (*Populus trichocarpa*). In the present study, we used the acetyl bromide method [63] to measure lignin in the association panel given that is faster, simpler and presents better recovery of lignin in different herbaceous tissues than Klason- [64] and thioglycolic acid-based methods [65]. In our GWAS, we identified seven QTL regions, with one of them (CH11) coinciding with the one found in the GWAS for digestibility. This is in contrast with the results of Penning et al**.,** in maize, where they did not find overlapping QTL for lignin abundance and saccharification [22]. This common QTL in CH11 contains a homolog of *HCT*. Although there are no reports published about functional studies of any *OsHCT*, in *Medicago, HCT* expression determines stem digestibility [47]. As well as candidates in monolignol synthetic pathways, some QTL contain putative candidate genes involved in lignin polymerization such as a cluster of seven peroxidase genes located next to the QTL peak on CH3 and a laccase gene in the QTL region CH11_18.8 ± 0.3. Homologues of these genes in Arabidopsis and tobacco are involved in determining lignin content [66–68].

## Conclusion

The use of crop residue biomass provides a way to avoid competition between biofuel and food production for feedstock. Since rice straw is an abundantly available and globally underutilized resource, it provides an attractive feedstock for bio-refining [69]. However, to take full advantage of this resource, we need to improve its processing potential and make it more easily digestible with industrial enzymes to allow the production of cost-competitive sustainable biofuels by fermentation. To this end, we have assembled a diversity panel from rice germplasms in Vietnam, which is the fourth largest rice exporter in the world [70]. Rice is a cereal with a small-sized diploid genome (~ 430 Mb), well-developed molecular genetics tools, and has representative cell wall characteristics of grasses, making it an important crop from which to extrapolate knowledge on cell wall to other cereals [71]. This is important because our understanding of the biosynthetic gene machinery and molecular structure of plant cell walls remains incomplete and the molecular basis of biomass digestibility even more so.

The availability of accurate genomic information in rice opens the possibility for precise and robust GWAS for multigenic traits such as saccharification. We produced a high-density SNP matrix for 151 rice cultivars that were in parallel phenotyped for straw digestibility and lignin content. We were able to identify a number of QTL for these parameters and proposed a number of candidate genes associated with some of these QTL. Besides these QTL, we could identify outstanding genotypes that can be included in breeding programs for biomass quality. The markers identified could be validated and used in a breeding program for the selection of high digestible straw genotypes with a potential increase of up to 48 kg ha^−1^ of sugar released (Additional file 4).

In conclusion, association mapping for two traits associated with rice straw quality succeeded in identifying genetic variation in genomic regions that contain plausible candidate genes affecting digestibility. This forward genetic approach is a powerful way to identify known and novel genes involved in these traits. Future work is nevertheless required to validate these candidates and carry out the functional studies required to confirm their roles in cell wall biosynthesis. Such validation will lead to the robust application of associated molecular markers in breeding programs aiming to select plants with improved digestibility and avoid grain yield penalties.

## Methods

### Mapping population

The association panel comprises 151 rice genotypes from Vietnam, which originated from two *Oryza sativa* subspecies: *indica* and *tropical japonica*. These genotypes were selected from a trial population derived from a breeding project at the Plant Biotechnology Division, Field Crops Research Institute (FCRI), 84 different genotypes which are reserved in the Germplasm Bank of FCRI, 29 high-quality genotypes which are popularly cultivated in different areas in Vietnam, and 38 landrace cultivars. These collected genotypes are expected to be highly inbred lines with homozygous genomic background. (See Additional file 5 for the list of the genotypes used). From these, a subset of 93 genotypes was grown in 2013 and the full panel was grown in 2014. Several field traits of this population from other trials such as plant height, flowering time, and grain yield are listed in Additional file 6.

The association panel was grown in the field, in Hai Duong province, the north of Vietnam (GPS coordinates are attached in Additional file 7). The first field trial, including 93 single plots, was sown in January and harvested in May 2013, and the second field trial, including 151 single plots, was sown in June and harvested in October 2014. Straw samples for each genotype were collected from five plants in the plot (plot size = 2 × 5 m = 10 m^2^, plant density/plot = 40/m^2^) after harvest for grain, and these five plants were kept separately as five replicates for each genotype. All samples were taken from the main tiller. The straw collected was dried for 2 days in the open air in Vietnam. Straw samples were kept in separate paper bags and sent to the Centre for Novel Agricultural Product (CNAP), University of York, UK, for characterization. The rice stems (minus nodes) were cut into small pieces, then ground to a fine powder and stored. These samples were used for different assays including saccharification, and total lignin content.

### Phenotyping for cell wall traits

#### Saccharification assay

The saccharification for 93 genotypes in 2013 and 151 genotypes in 2014 was analyzed using an automated platform as described in Gomez et al. [52]. Samples of five plants from the same genotypes were treated as five separated replicates. In brief, ground straw samples were formatted in 96 well plates, in randomized positions, with four technical replicates of 4 mg for each sample using a robotic platform (Labman Automation, Stokesley, North Yorkshire, UK) [39]. The samples were analyzed using a liquid handling robot (Tecan LTD, UK), which performed a water pre-treatment at 94 °C for 20 min, followed by an enzymatic hydrolysis during 8 h at 50 °C. The enzyme used for saccharification was a 4:1 mixture of Celluclast and Novozyme 188 (Novozymes). The saccharification was estimated by measuring the reducing sugars released from the biomass material. This was done with a colorimetric assay using 3-methy-2-benzothiazolinone hydrazone method (MBTH) [39, 52]. Three standards of 50, 100 and 150 nmol glucose (three replicates each) and filter paper disks (four replicates)—as control—were used to account for any change in enzyme concentration or condition through time.

#### Total lignin content

Lignin content was quantified using acetyl bromide [72]. Three replicates from each straw sample were used for lignin determination. Four mg of ground samples was weighed into 2 ml tubes and 250 µl freshly prepared acetyl bromide solution (25% v/v acetyl bromide/75% glacial acetic acid) was added before incubating at 50 °C for 2 h, followed by a further 1 h with vortexing every 15 min to solubilize the lignin. Samples were then cooled to room temperature before being transferred to 5 ml volumetric flasks. Subsequently, 1 ml of 2 M NaOH was added, followed by 175 µl freshly prepared 0.5 M hydroxylamine hydrochloride. After shaking, the samples were then made up to 5 ml with glacial acetic acid, and the 280 nm absorbance was read using a Shimadzu UV-1800 spectrophotometer. Lignin content (µg.mg-1 cell wall) was determined using the following formula: (Absorbance ÷ (coefficient × path length)) × ((total volume × 100%) ÷ biomass weight)). The coefficient for grass (17.75) was used for rice [72].

#### Data analysis

The analysis of the raw saccharification and lignin content data took into account sources of non-genetic variation relating to field and laboratory factors [33]. The genotype means used in GWAS are therefore adjusted rather than raw means. All statistical analysis were obtained from using R-package asreml (https://www.vsni.co.uk/software/asreml-r) in R studio (https://www.rstudio.com/). To avoid the population structure misleading GWAS analysis, we decided to remove the *japonica* subpopulation. The trait file of *indica* genotype used in GWAS is listed in Additional file 8.

### Genotyping data

The genotypic data was produced by genotyping by sequencing (GBS) assays. 172 rice genotypes were sequenced on an Illumina platform at the Rice Laboratory, Cornell University, USA. The GBS assay involved library construction, sequencing, data analysis, and SNP detection from HapMap, following the methods described in [73]. The GBS analysis pipeline (Tassel Version: 3.0.166, date: April 17, 2014) was applied to analyze the data after sequencing [74]. The report of the GBS is attached as Additional file 9.

### Population stratification using GAPIT

To study stratification of the population, a phylogenetic tree was created from GAPIT (Fig. 2) [37, 38]. This was determined based on the kinship matrix, which accounts for the degree of genetic relatedness or coefficient of relationship between individual members of the population. Kinship among genotypes was calculated using an R implementation (www.R-project.org) available as part of GAPIT software libraries [38, 75]. Using output distances, clustering was performed in R using the internal package “hclust” with default parameters.

### Mixed linear model (MLM) using tassel

Based on the genotypic data stored in the HapMap and the phenotypic data collected from the analysis of saccharification from 2013 and 2014 harvest (sugar released) and lignin content from 2014 harvest (% of total lignin), GWAS was performed by merging genotype and each phenotype to examine the association between the markers and the studied trait to identify the quantitative trait loci (QTL).

GWAS was performed using the compressed mixed linear model approach, which includes both fixed and random effects [37, 76] carried out by TASSEL [41] that was also implemented in the Efficient Mixed-Model Association (EMMA) [77] for performing association mapping while simultaneously correcting for relatedness and population structure.

The data were merged and manipulated with Tassel 3.0 [41]. The Q Matrix file was created, using PSIKO (https://www.uea.ac.uk/computing/pisko) on a Linux platform. The proportion of the phenotypic variation explained (PVE) by each marker was estimated by the relevant R2 in TASSEL [41, 78].

The significant level for association with a SNP in the Fig. 7 was based on FDR value. Please find the formula for calculating FDR as follows. FDR = *p*value × (*n*/rank), in which *n* = total number of SNP, and rank = ranking of SNP based on *p *value. FDR < 0.05 = significant (−log 10 of the last significant value = 5% FDR cutoff).

## Supplementary information

**Additional file 1.** Containing Q-Q plots of GWAS

**Additional file 2.** Containing the list of genes locate in the regions of digestibility QTL

**Additional file 3.** Containing the list of genes locate in the regions of Lignin QTL

**Additional file 4.** Detailed calculation for estimated gains of using a marker in breeding

**Additional file 5.** Containing the list of rice line genotypes from GWAS population

**Additional file 6.** Agronomic trait data of sequenced genotypes

**Additional file 7.** Field trial GPS coordinates

**Additional file 8.** Containing data of studied traits

**Additional file 9.** Containing a report of Genotyping by Sequencing (GBS) – Reference Pipeline

## Data Availability

All supporting data are provided with this submission and additional files are detailed below.

## Abbreviations

4CL: Hydroxycinnamate-CoA ligase
ANOVA: Analysis of variance
AM: Association mapping
BAHD: Superfamily named after the first four members of the family to be biochemically characterized (BEAT: benzylalcohol acetyltransferases, AHCT: anthocyanin hydroxycinnamoyl transferase, HCBT: anthranilate hydroxycinnamoyl/benzoyl transferase, DAT: Deactylvindoline acetyltransferase)
CCR: Cinnamoyl-CoA-reductase
CH: Chromosome
FA: Ferulic acid
FDR: False discovery rate
GBS: Genotyping by sequencing
GWAS: Genome-wide association study
GT: Glycosyltransferase
HCT: Hydroxycinnamoyl-CoA shikimate/quinate transferase
*irx*: Irregular xylem mutant
LD: Linkage disequilibrium
LOD: Logarithm of the odds
MBTH: 3-Methy-2 benzothiazolinone hydrazone
MLM: Mixed linear model
p-CA: P-Coumaric acid
PCA: Principal component analysis
PCC: Pearson correlation coefficient
PVE: Phenotypic variance explained
QTL: Quantitative trait loci
RIL: Recombinant inbred lines
SNP: Single nucleotide polymorphism
SSR: Simple sequence repeat
