# Supplementary figures and images for: Association mapping identifies quantitative trait loci (QTL) for digestibility in rice straw
Source: Biotechnol Biofuels. 2020 Oct 8;13:165. doi: 10.1186/s13068-020-01807-8 (PMC7545568; doi:10.1186/s13068-020-01807-8)

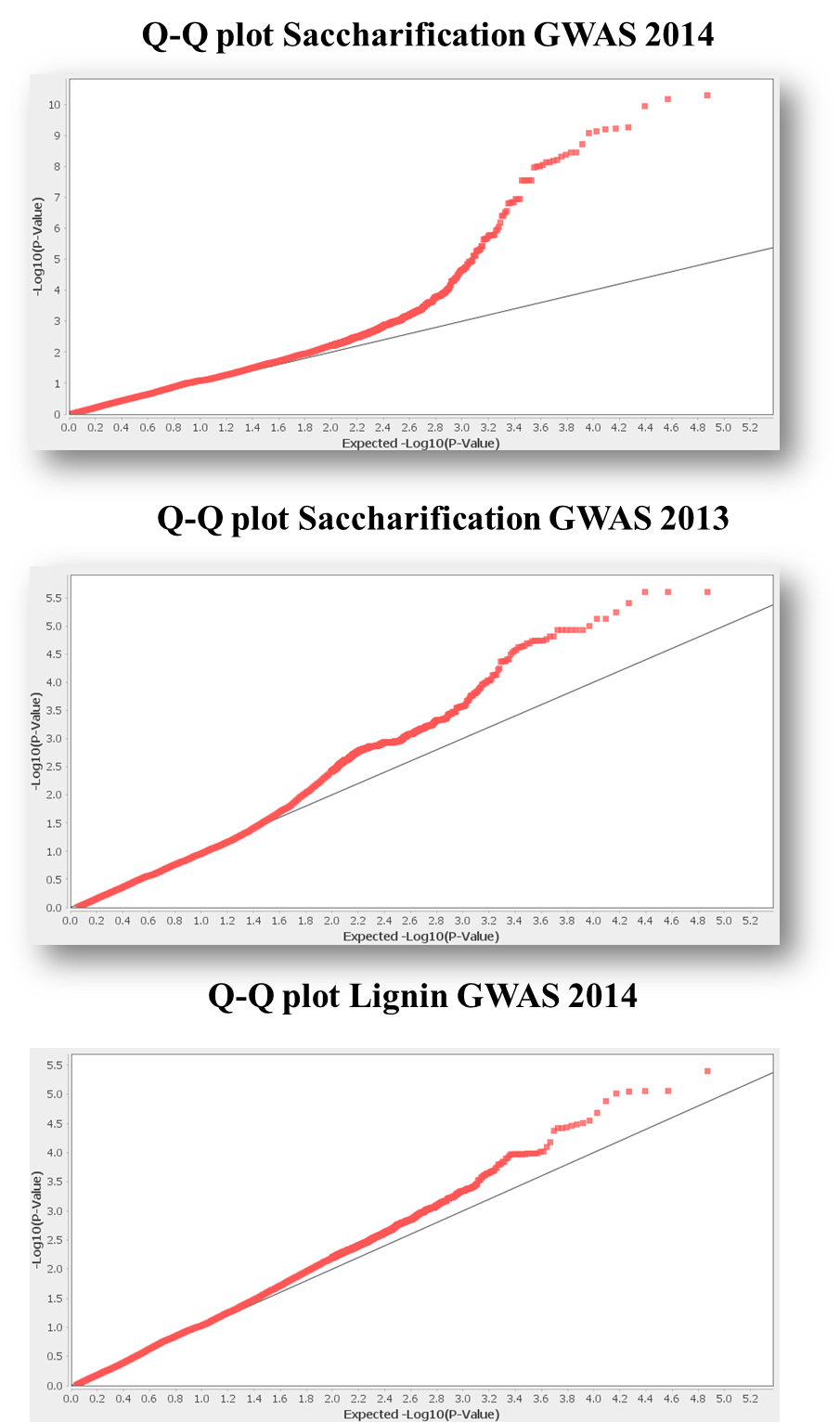

Supplement: Supplementary file 1 — Additional file 1. Containing Q-Q plots of GWAS [file 13068_2020_1807_MOESM1_ESM.docx]
