# Supplementary material for: Association mapping identifies quantitative trait loci (QTL) for digestibility in rice straw
Source: Biotechnol Biofuels. 2020 Oct 8;13:165. doi: 10.1186/s13068-020-01807-8 (PMC7545568; doi:10.1186/s13068-020-01807-8)
Supplement: Supplementary file 4 — Additional file 4. Detailed calculation for estimated gains of using a marker in breeding [file 13068_2020_1807_MOESM4_ESM.docx]

*What would the gain in sugar-release be per hectare if some of the markers identified were used in a breeding program?*

Based on the proportion of phenotypic variation explained (PVE) by significant markers (SNPs), that was estimated by relevant markerR^2^, we can calculate sugar-release gained per hectare if a marker was included in a breeding program as follows:

The mean of straw yield at harvest was 2.54 Mgdw ha−1 (2.54 tons/hectare), reported by Nguyen-Van-Hung et al., “An assessment of irrigated rice production energy efficiency and environmental footprint with in-field and off-field rice straw management practices”, Scientific Reports volume 9, Article number: 16887 (2019). SNP at peak on CH7 (29.4 Mb) explained 36%. A genotype with an average value of sugar release carrying the non-favorable allele of this CH7 QTL in our population could generate 47.9 nmol/mg.1hour.

47.9.3 nmol/mg.1hour => 383 nmol/mg.8hour => 383mol/tons => 383*2.54 972mol/hectare (~160kg ha^-1^). When this genotype is crossed and selected with favourable allele that is associated with CH7 QTL/causative gene(s), the selected recombinant line could gain 36% in sugar release, which is equal to (972 * 30/100 = 291mol/hectare = 48kg ha^-1^).
