# Supplementary material for: Association mapping identifies quantitative trait loci (QTL) for digestibility in rice straw
Source: Biotechnol Biofuels. 2020 Oct 8;13:165. doi: 10.1186/s13068-020-01807-8 (PMC7545568; doi:10.1186/s13068-020-01807-8)
Supplement: Supplementary file 7 — Additional file 7. Field trial GPS coordinates [file 13068_2020_1807_MOESM7_ESM.docx]

**Field Trial location of association mapping population in 2013 and 2014**


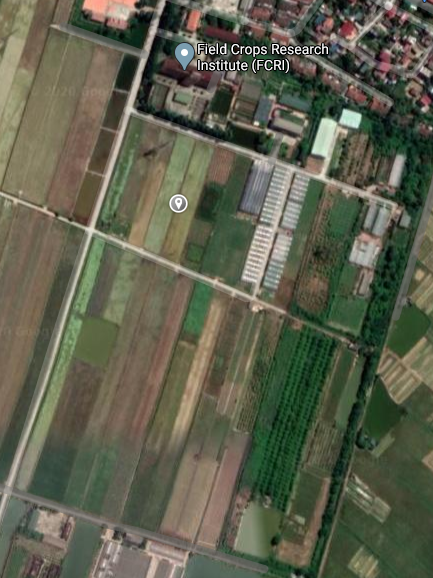


| 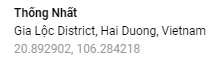 | 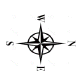 |
| --- | --- |

|  | Rice buffer |  |
| --- | --- | --- |
| Rice buffer | Experimental plots (93 plots in 2013 and 151 plots in 2014)  (plot size = 2 x 5m = 10m^2^, plant density/plot = 40/m^2^) | Rice buffer |
|  | Rice buffer |  |
