# Supplementary material for: Association mapping identifies quantitative trait loci (QTL) for digestibility in rice straw
Source: Biotechnol Biofuels. 2020 Oct 8;13:165. doi: 10.1186/s13068-020-01807-8 (PMC7545568; doi:10.1186/s13068-020-01807-8)
Supplement: Supplementary file 9 — Additional file 9. Containing a report of Genotyping by Sequencing (GBS) – Reference Pipeline [file 13068_2020_1807_MOESM9_ESM.pdf]

Biofuels Rice McCouch

Genotyping by Sequencing (GBS) Reference Pipeline

Prepared by Katie Hyma

2/4/2015

For questions regarding your project/data specifically please contact gbsdata@cornell.edu and keh233@cornell.edu

For general questions regarding the GBS pipeline, GBS data analysis and TASSEL, please use the TASSEL google group here, so that others may benefit from your questions and answers: https://groups.google.com/forum/?fromgroups#!forum/tassel

additional GBS resources are provided below

Please refer to the Institute for Biotechnology's guidelines for acknowledgement and authorship: http://www.biotech.cornell.edu/brc/brc/services/terms-and-policies

Contents

Sample Details.......... 2
Analysis Notes.......... 2
Overview of the Analysis Pipeline.......... 3
Description of Data Files.......... 4
HapMap files.......... 4
VCF files.......... 5
TOPM file:.......... 6
Additional files:.......... 7
GBS reference pipeline options used for analysis.......... 8
Reads and Tags.......... 10
Summary of Reads and Tags found in each Sequencing Lane.......... 10
Total number of Tags after Merging.......... 10
Number of Reads per Individual and Failed Samples.......... 10
Genome Info.......... 17
BWA Alignment parameters.......... 17
Alignment Results:.......... 17
Resulting SNPs.......... 18
Multi Dimension Scaling (MDS) of genome-wide SNPs from the reference genome pipeline:.......... 19
Genotyping by Sequencing Resources.......... 21
GBS Overview.......... 21
GBS Frequently Asked Questions.......... 21
GBS Bioinformatics.......... 21
Training.......... 21
TASSEL.......... 21
Bibliography:.......... 22

Sample Details

175 uniquely named samples (190 total) and 2 blank(s) digested with enzyme(s) ApeKI

Library Plate	Library Plate ID	Filename (Flowcell_lane_fastq.gz)	Md5sum
Biofuel_Rice_p1	450021747	C5W6FACXX_5_fastq.gz	27358626d9953ffbe93cb5f9aef578a6
Biofuel_Rice_p2	450021748	C5W6FACXX_6_fastq.gz	21334921fd284ac9f7c1333a07fed6d9

Analysis Notes

The GBS analysis pipeline (Tassel Version: 3.0.166 Date: April 17, 2014) was run on these samples

If appropriate, VCF snps were merged with Tassel Version: 4.3.7 Date: April 17, 2014) – see description of VCF files.

An overview of GBS and the GBS pipeline can be found here: http://www.maizegenetics.net/Table/Genotyping-By-Sequencing/. See the following sections for a list of options used for this analysis.

We are providing:

1) SNP calls output from the GBS Bioinformatics pipeline (see description of data files below).
2) TOPM (tags-on-physical-map) file (see description of data files below
3) Keyfile(s) used to associate barcodes with sample IDs while running the GBS pipeline.
4) This report summarizing the SNP calling procedure, including software versions used
5) XML configuration files that can be used (with the software versions indicated) to reproduce the results summarized here. XML files can be used by invoking the corresponding TASSEL plugin in the following manner (adjusting the maximum amount of memory –Xmx if necessary)

    run_pipeline.pl –Xmx8G –configFile xmlfile.xml

    as directory structures and file locations differ, please edit the corresponding options appropriately if you wish to utilize the .xml file for your own analysis.

***Please note,** the GBS pipeline is continually being developed, and as such the results from this run can only be reproduced with the GBS pipeline version noted above, along with the options listed in the following section or utilizing the xml files distributed with your data.

Overview of the Analysis Pipeline

The reference-based pipeline is the ‘discovery’ pipeline described in TASSEL 3.0 documentation and also in Glaubitz et al. 2013.

Please refer to the TASSEL 3.0 documentation for a full description of all Plugins:
https://bytebucket.org/tasseladmin/tassel-5-source/wiki/docs/TasselPipelineGBS.pdf

<u>Discovery Pipeline Overview</u>

The flow chart below shows how the steps of a possible GBS “Discovery Pipeline” analysis link together (variations on this approach are possible). Light blue boxes represent files (or data structures) produced at each step of the analysis, and purple boxes represent the processes (Tassel3 plugins) that produced them:

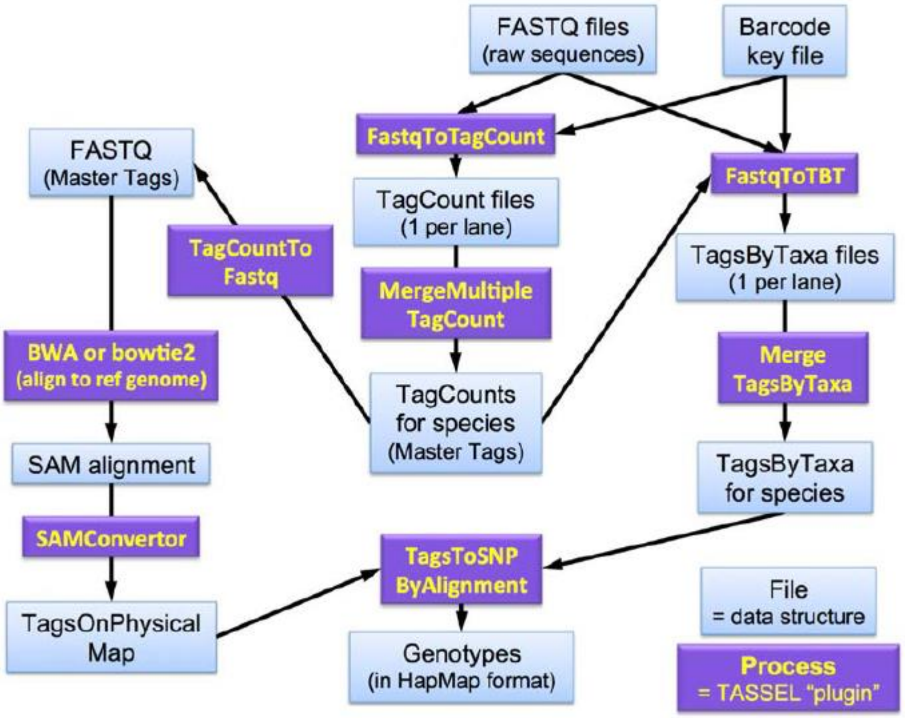

Description of Data Files

We are providing SNP calls in both HapMap and VCF format. Currently SNP calling as implemented for hapmap and VCF files are independent, and as such variations between the two files can be expected.

HapMap files

HapMap is the standard file format generated by the TASSEL GBS pipeline. We are providing two sets of HapMap files:

1. A set without post SNP-calling filtering (located in the folder "hapmap/unfiltered")
2. A set with additional filtering on missingness and allele frequency (located in the folder "hapmap/filtered).

Within both folders you will find either one or two sets of files, depending on whether your project included identically named samples.

1. A hapmap file with separate SNP calls for identically named taxa (c+.snpmerged.hmp.txt)
2. A hapmap file with SNPs merged for identically named taxa. (c+.taxaMerged.hmp.txt)

The naming convention of these HapMap files is c+, where + is the chromosome number (where available). In the case of an assembly that is not in the chromosome/pseudomolecule stage there will be one or more files, but "+" will not correspond to a chromosome number. Columns 3 and 4 in the HapMap file correspond to chromosome (or contig/scaffold) and position, respectively.

For GBS libraries sequenced multiple times, identical samples are merged prior to SNP calling. Identically named samples that did not originate from the same library are merged after SNP calling.

Here we have filtered on missingness only (see the options listed below for the plugin GBSHapMapFiltersPlugin). We strongly recommend filtering your data with parameters that are appropriate for your species/project. HapMap files can be easily opened and filtered in TASSEL 5.0. http://www.maizegenetics.net/index.php?option=com_content&task=view&id=89&Itemid=119

The header line of the hapmap file describes the information contained within.

VCF files

VCF is an alternative format for holding SNP information that retains information on depth of coverage for each allele, and can be output from the GBS pipeline by replacing the plugins 'TagsToSNPByAlignmentPlugin' and 'MergeDuplicateSNPsPlugin' with 'tbt2vcfPlugin' and 'MergeDuplicateSNP_vcf_Plugin'. Genotype likelihood scores are calculated based on formula 3.8 of Etter et al 2013=1, and the most likely genotype is assigned. Genotype quality (GQ) score is calculated to the GATK version documented here: http://gatkforums.broadinstitute.org/discussion/1268/how-should-i-interpret-vcf-files-produced-by-the-gatk . The VCF format is not currently supported for downstream analysis with TASSEL 3.0, although support for VCF in TASSEL 4.0 and TASSEL 5.0 has now been implemented.

We are providing one file (all.mergedSNPs.vcf.gz) that contains all SNP calls, and has been compressed with bgzip. You can unzip this file with the command gunzip on Linux/Unix, or with any of several software options on windows (7-zip is a good one).

The header lines of this file (beginning with #) contain information regarding the contents of the file. Please note that the alleles are in the format major/minor rather than reference/alternate.

Multiply sequenced or identically named samples are not merged during VCF SNP calling, but identically named samples are merged after SNP calling with the experimental **TASSEL 4.0 plugin "MergeIdenticalTaxaPlugin"** as a courtesy. The xml file for this plugin is included in the data distribution.

You can also work with VCF files output from the GBS pipeline using VCFtools (http://vcftools.sourceforge.net/) and other software platforms. VCFtools is currently only available for Linux/Unix platforms. If you do not have access to a Linux computer and wish to use VCFtools, you can purchase machine hours on CBSU's BioHPC Computing Lab (http://cbsu.tc.cornell.edu/lab/Pricing.aspx).

For heterozygous materials and diversity studies (in cases where there is little information at the population level to leverage for filtering SNPs), and especially where sequence coverage is low (as is typical for GBS data), we strongly suggest working with the VCF files and utilizing the allelic read depth and/or SNP quality information for filtering.

TOPM file:

This file contains the actual tag sequences (each unique 64-bp sequence that was present across all samples) and tag alignment information. It is a TOPM "tags on physical map" file.

You will find:

In the header line, listed by column:

1) number of tags in the file,
2) number of “longs” used to store a tag. 1 long = 32 bp. 2 longs = 64 bp.
3) Maximum number of variants stored per tag (meaningless for this SNP discovery pipeline)

In each subsequent line, listed by column:

1) The tag sequence
2) The length of the tag (tags shorter than 64 bp are padded with “A” in this file, but the extra padding is not used during SNP calling
3) The number of genomic positions the tag aligns to (99 means multiple >1 positions).
4) Chromosome (* means no unique alignment position)
5) Strand (1 or -1, * means no unique alignment position) [note: for tags that align to a unique position but on the minus strand End Position < Start Position]
6) Start Position of the barcoded end (* means no unique alignment position)
7) End Position of the common adapter end, (* means no unique alignment position)
8) Divergence (edit distance from the aligned position on the reference genome).
9) The remainder of each row consists of position offsets and the alleles for the 8 variants. They are meaningless here, and will all be “*”

You can use the SNP chromosome and position from the hapmap (columns 3 and 4) or VCF file (columns 1 and 2) in conjunction with the tag start and stop positions in the TOPM file to find tag sequences contributing to the locus.

Additional files:

Please note that if your genome was "concatenated" into fake chromosomes, we have also included an index file (projectname.sam.index) that relates the original coordinates to the fake "pseudochromosome" coordinates in the form as follows, where realchr# is the name of the original scaffold/contig/chromosome from the reference fasta file described in the reference genome section of this report.

Fakechr# start length realchr#

For your convenience, the final SNP calls and TOPM file have been "de-indexed" so that the chromosome and position coordinates relate to the original genome file, with chromosome names changed as needed for compatibility with TASSEL (see genome info section for specifics).

If you are repeating the analysis and want to follow this procedure yourself, you will need to use the scripts publicly available at https://bitbucket.org/khyma/igd_public

You would use the script "concatenate.pl" with the .sam output from the aligner as input, and then use the result (a modified .sam file) with the TASSEL SAMConverterPlugin to create a "TOPM" file with the fake chromosomal coordinates and continue with the pipeline from there. This script also produces the index file referenced above.

After calling SNPs, the script "deconcatenate.pl" can be used with a hapmap, vcf, or a text topm file (to convert a binary TOPM file to text TOPM, so will need to use TASSEL's BinaryToTextPlugin).

These scripts are written in perl and assume that you are running in an environment where perl is installed and system calls to the bash functions head/tail/grep/sort/cat are available. If you run each of these perl scripts without any options they will print the usage statement describing expected inputs.

GBS reference pipeline options used for analysis

(not including file direction options – please see xml files for full list of options used)

Plugin	Option	Value	Description
FastqToTagCountPlugin	c	1	Minimum number of times a tag must be present to be output. Default: 1
FastqToTagCountPlugin	s	300000000	Max good reads per lane. (Optional. Default is 300000000).
MergeMultipleTagCountPlugin	c	3	Minimum number of times a tag must be present to be output. Default: 1
TagCountToFastqPlugin	c	1	Minimum count of reads for a tag to be output (default: 1)
FastqToTBTPlugin	y	-y	output to tagsByTaxaByte (tag counts per taxon from 0 to 127) instead of tagsByTaxaBit (0 or 1)
FastqToTBTPlugin	c	1	Minimum taxa count within a qseq file for a tag to be output. Default: 1
MergeTagsByTaxaFilesPlugin	s	300000000	Maximum number of tags the TBT can hold while merging (default: 200000000)
TagsToSNPByAlignmentPlugin	y	-y	Use byte-formatted TBT file (*.tbt.byte)
TagsToSNPByAlignmentPlugin	errRate	0.01	Average sequencing error rate per base (used to decide between heterozygous and homozygous calls) (default: 0.01)
TagsToSNPByAlignmentPlugin	mnLCov	0.1	Minimum locus coverage i.e. the proportion of taxa with at least one tag at the locus. Default: 0.1
TagsToSNPByAlignmentPlugin	mxSites	2000000	The maximum number of SNPs per chromosome for hapmap files (default = 2000000)
TagsToSNPByAlignmentPlugin	mnMAC	999	Minimum minor allele count. Defaults to 10. SNPs that pass either the specificed minimum minor allele count (mnMAC) or frequency (mnMAF) will be output.
TagsToSNPByAlignmentPlugin	mnMAF	0.01	Minimum minor allele frequency. Defaults to 0.01. SNPs that pass either the specifice dminimum minor allele frequency (mnMAF) or count (mnMAC) will be output.
MergeDuplicateSNPsPlugin	misMat	0.05	Threshold mismatch rate above which the duplicate SNPs won't be merged. Default: 0.05.
MergeDuplicateSNPsPlugin	callHets	-callHets	When two genotypes at a replicate SNP disagree for a taxon call it a heterozygote. Defaults to false (=set to missing)

Plugin	Option	Value	Description
MergeIdenticalTaxaPlugin	hetFreq	0.8	cutoff frequency between het vs. homozygote calls (default = 0.8)
FastqToTBTPlugin	y	-y	output to tagsByTaxaByte (tag counts per taxon from 0 to 127) instead of tagsByTaxaBit (0 or 1)
FastqToTBTPlugin	c	1	Minimum taxa count within a qseq file for a tag to be output. Default: 1
MergeTagsByTaxaFilesPlugin	s	300000000	Maximum number of tags the TBT can hold while merging (default: 200000000)
tbt2vcfPlugin	ak	3	Maximum number of alleles that are kept for each marker across the population default: 3
tbt2vcfPlugin	mnLCov	0.0	Minimum locus coverage (proportion of Taxa with a genotype) (default: 0.0)
tbt2vcfPlugin	mnMAF	0.0	Minimum minor allele frequency (default: 0.0)
MergeDuplicateSNP_vcf_Plugin	ak	3	Maximum number of alleles that are kept for each marker across the population default: 3
GBSHapMapFiltersPlugin	mnSCov	0.8	Minimum site coverage (default: no filter)
GBSHapMapFiltersPlugin	mxMAF	1	Maximum minor allele frequency (default: 1.0 = no filter)
GBSHapMapFiltersPlugin	mnTCov	0.1	Minimum taxa coverage (default: no filter)
GBSHapMapFiltersPlugin	mnMAF	0.01	Minimum minor allele frequency (default: 0.0 = no filter)

Reads and Tags

Summary of Reads and Tags found in each Sequencing Lane

**Please note that blanks must be named “blank,” case insensitive to be automatically excluded from summary calculations **

FastQ file	Barcodes found in lane	Total # of reads per lane	Total number of good barcoded reads	Resulting # of Tags
C5W6FACXX_5_fastq.gz	96	229727166	207579114	25375094
C5W6FACXX_6_fastq.gz	96	231492333	220050998	18824732

Total number of Tags after Merging

- 4609509

Number of Reads per Individual and Failed Samples

Failed samples (non-blank) are defined as those with less than 10% of the mean reads per sample coming from the lane on which they were sequenced.

Total number of samples: 192
Total number of failures: 0 (0.00%)

Flowcell	lane	Barcode	ID	good barcoded reads	plate	row	col	Fail?
C5W6FACXX	5	AGGC	BB1_11	2586074	Biofuel_Rice_p1	A	1	pass
C5W6FACXX	5	GATT	U17	2465797	Biofuel_Rice_p1	A	2	pass
C5W6FACXX	5	ACCGT	OM6613	2244248	Biofuel_Rice_p1	A	3	pass
C5W6FACXX	5	CGTCA	OM4325	3272765	Biofuel_Rice_p1	A	4	pass
C5W6FACXX	5	TCGCA	Beo_Buot_Vang	2028665	Biofuel_Rice_p1	A	5	pass
C5W6FACXX	5	CGCAT	AC5_149_13	1930680	Biofuel_Rice_p1	A	6	pass
C5W6FACXX	5	TCATAGT	Lua_Thom	1924075	Biofuel_Rice_p1	A	7	pass
C5W6FACXX	5	TTACGAT	X_mai	2019827	Biofuel_Rice_p1	A	8	pass
C5W6FACXX	5	GGCTAGA	Caren	3445889	Biofuel_Rice_p1	A	9	pass
C5W6FACXX	5	ACAATGGA	AC5_Q5__AC4	2820571	Biofuel_Rice_p1	A	10	pass
C5W6FACXX	5	ACAAGAGT	CL8_AC5	2185161	Biofuel_Rice_p1	A	11	pass
C5W6FACXX	5	GAACATGA	D381_Q5	2628275	Biofuel_Rice_p1	A	12	pass
C5W6FACXX	5	AGCATT	BB10	2423007	Biofuel_Rice_p1	B	1	pass
C5W6FACXX	5	CTCCGA	KD18	2693618	Biofuel_Rice_p1	B	2	pass
C5W6FACXX	5	TTGGCA	A__hung_cha	2622644	Biofuel_Rice_p1	B	3	pass
C5W6FACXX	5	CCACGT	RD354	1996567	Biofuel_Rice_p1	B	4	pass

Flowcell	lane	Barcode	ID	good barcoded reads	plate	row	col	Fail?
C5W6FACXX	5	GATGTC	142M12	1923429	Biofuel_Rice_p1	B	5	pass
C5W6FACXX	5	TGTTAC	HDT4	1629565	Biofuel_Rice_p1	B	6	pass
C5W6FACXX	5	CAGTTA	P13	2156848	Biofuel_Rice_p1	B	7	pass
C5W6FACXX	5	GCCTAT	S_c_tr_ng	1923568	Biofuel_Rice_p1	B	8	pass
C5W6FACXX	5	AGTGGC	Khau_mu_lai_dong_1	1193679	Biofuel_Rice_p1	B	9	pass
C5W6FACXX	5	TGACCT	A__hung_cha_2	2007243	Biofuel_Rice_p1	B	10	pass
C5W6FACXX	5	TTGCAC	DT7_LT2__BT7	1657864	Biofuel_Rice_p1	B	11	pass
C5W6FACXX	5	CTAGCT	BT_ST	1695026	Biofuel_Rice_p1	B	12	pass
C5W6FACXX	5	AATCGTT	BB5_10	2207646	Biofuel_Rice_p1	C	1	pass
C5W6FACXX	5	CTATGGA	SH4	2475210	Biofuel_Rice_p1	C	2	pass
C5W6FACXX	5	TACGGTA	Kham_Duc	2316457	Biofuel_Rice_p1	C	3	pass
C5W6FACXX	5	ACTATGT	LT2	1430281	Biofuel_Rice_p1	C	4	pass
C5W6FACXX	5	CGTGAAT	WC5_AC5__KD18	1937386	Biofuel_Rice_p1	C	5	pass
C5W6FACXX	5	TTGCAGA	AC5_HT1	2259240	Biofuel_Rice_p1	C	6	pass
C5W6FACXX	5	AACTTGT	Nghi_Huong	1791769	Biofuel_Rice_p1	C	7	pass
C5W6FACXX	5	TGACGTA	BB3	2074702	Biofuel_Rice_p1	C	8	pass
C5W6FACXX	5	GCTATAA	AC5_Q5__AC4	2317385	Biofuel_Rice_p1	C	9	pass
C5W6FACXX	5	ATCGTAT	Perai_BT7	1741802	Biofuel_Rice_p1	C	10	pass
C5W6FACXX	5	TACTGAT	OM5451	2043422	Biofuel_Rice_p1	C	11	pass
C5W6FACXX	5	CTTGAGA	OM2517	2542837	Biofuel_Rice_p1	C	12	pass
C5W6FACXX	5	TCAAGTT	IR24	2228747	Biofuel_Rice_p1	D	1	pass
C5W6FACXX	5	GATCATA	Q5	2230717	Biofuel_Rice_p1	D	2	pass
C5W6FACXX	5	GCATTGA	OM9218	2480369	Biofuel_Rice_p1	D	3	pass
C5W6FACXX	5	CAGGTAT	OM5930	1206603	Biofuel_Rice_p1	D	4	pass
C5W6FACXX	5	TGCAATA	AC10_Fukus	2315570	Biofuel_Rice_p1	D	5	pass
C5W6FACXX	5	ATATCGT	TL6	1713227	Biofuel_Rice_p1	D	6	pass
C5W6FACXX	5	AGTCTAT	N46	1614625	Biofuel_Rice_p1	D	7	pass
C5W6FACXX	5	GTCTGAA	CL8_P6	2205307	Biofuel_Rice_p1	D	8	pass
C5W6FACXX	5	ATCAGTT	BT7_LT2	1571921	Biofuel_Rice_p1	D	9	pass
C5W6FACXX	5	CAGTTGA	AC5	2263191	Biofuel_Rice_p1	D	10	pass
C5W6FACXX	5	TGTGCAA	BB1_10_LT2	2176617	Biofuel_Rice_p1	D	11	pass
C5W6FACXX	5	CGACAGT	N46__B6	1759755	Biofuel_Rice_p1	D	12	pass
C5W6FACXX	5	ACGTGTA	BB21	3025538	Biofuel_Rice_p1	E	1	pass
C5W6FACXX	5	GATGCAT	P6	1849543	Biofuel_Rice_p1	E	2	pass
C5W6FACXX	5	CTAATGT	OM68__	1694700	Biofuel_Rice_p1	E	3	pass

Flowcell	lane	Barcode	ID	good barcoded reads	plate	row	col	Fail?
C5W6FACXX	5	GTCGATA	N91	2302390	Biofuel_Rice_p1	E	4	pass
C5W6FACXX	5	TATACGT	Q5_Fukus__Q5	1560278	Biofuel_Rice_p1	E	5	pass
C5W6FACXX	5	GCGTAAT	IR_	1735311	Biofuel_Rice_p1	E	6	pass
C5W6FACXX	5	AGCGTTA	LT3	2543013	Biofuel_Rice_p1	E	7	pass
C5W6FACXX	5	ATCCGGA	Lua_nuong	2385027	Biofuel_Rice_p1	E	8	pass
C5W6FACXX	5	TCAGTAT	Perai_P6__HT1	1705787	Biofuel_Rice_p1	E	9	pass
C5W6FACXX	5	CAATGTT	BT__B5	1956306	Biofuel_Rice_p1	E	10	pass
C5W6FACXX	5	GTTACGA	IR1561__B__	2531592	Biofuel_Rice_p1	E	11	pass
C5W6FACXX	5	TGCATAT	OM4900	1639878	Biofuel_Rice_p1	E	12	pass
C5W6FACXX	5	CAAGAAGT	BB7	2785778	Biofuel_Rice_p1	F	1	pass
C5W6FACXX	5	GTCATGGT	SH4	2257972	Biofuel_Rice_p1	F	2	pass
C5W6FACXX	5	AACAGTGA	TET4247	2489737	Biofuel_Rice_p1	F	3	pass
C5W6FACXX	5	GTGCAAGA	Khau_mumeeng	2651911	Biofuel_Rice_p1	F	4	pass
C5W6FACXX	5	CAATAGGA	MT6	2085135	Biofuel_Rice_p1	F	5	pass
C5W6FACXX	5	TGCAGTGT	Lua_nuong	1813494	Biofuel_Rice_p1	F	6	pass
C5W6FACXX	5	AGGCTAGA	BTS7	2288041	Biofuel_Rice_p1	F	7	pass
C5W6FACXX	5	CTAGTGGT	AC5_Q5__C70	1680460	Biofuel_Rice_p1	F	8	pass
C5W6FACXX	5	GCTAGTGT	AC5_Q5__C70	1729912	Biofuel_Rice_p1	F	9	pass
C5W6FACXX	5	AGTTGGCA	BT_ST	2657083	Biofuel_Rice_p1	F	10	pass
C5W6FACXX	5	TCGCAAGT	Tan_nhe	2049196	Biofuel_Rice_p1	F	11	pass
C5W6FACXX	5	CGATGTGT	Jasmin_AC5	1912356	Biofuel_Rice_p1	F	12	pass
C5W6FACXX	5	AACGTAGA	Xi23	3564687	Biofuel_Rice_p1	G	1	pass
C5W6FACXX	5	CTCACGGA	Tran_chau_huong	2059524	Biofuel_Rice_p1	G	2	pass
C5W6FACXX	5	TAGCGTGT	OM5494	1797300	Biofuel_Rice_p1	G	3	pass
C5W6FACXX	5	ACGTAAGA	Khau_mumoong	2276046	Biofuel_Rice_p1	G	4	pass
C5W6FACXX	5	CGTATGGT	X26	1988048	Biofuel_Rice_p1	G	5	pass
C5W6FACXX	5	GTACGTGT	IR_	1696848	Biofuel_Rice_p1	G	6	pass
C5W6FACXX	5	TTCGAAGA	Que_Thom	2280579	Biofuel_Rice_p1	G	7	pass
C5W6FACXX	5	AATACGGA	Unknown	2099089	Biofuel_Rice_p1	G	8	pass
C5W6FACXX	5	TGACTGGT	P6_ofodiki__P6	2039588	Biofuel_Rice_p1	G	9	pass
C5W6FACXX	5	GCGGATGT	N46__B6	2046478	Biofuel_Rice_p1	G	10	pass
C5W6FACXX	5	CATTGAGA	BT_LT2	2498771	Biofuel_Rice_p1	G	11	pass
C5W6FACXX	5	GTAACAGA	OM5451	2308859	Biofuel_Rice_p1	G	12	pass
C5W6FACXX	5	AGCTTGGT	HT1	3048296	Biofuel_Rice_p1	H	1	pass
C5W6FACXX	5	ACAGATGA	Nep_98	3351631	Biofuel_Rice_p1	H	2	pass

Flowcell	lane	Barcode	ID	good barcoded reads	plate	row	col	Fail?
C5W6FACXX	5	CAGTTGGT	Tam_du	1847642	Biofuel_Rice_p1	H	3	pass
C5W6FACXX	5	TGCAAGAA	Khau_munuong	2424867	Biofuel_Rice_p1	H	4	pass
C5W6FACXX	5	ACTCGAGA	AC5	2575034	Biofuel_Rice_p1	H	5	pass
C5W6FACXX	5	GGAGCTGT	Nghi_Huong	2318625	Biofuel_Rice_p1	H	6	pass
C5W6FACXX	5	CTGAGTGT	D139	1780793	Biofuel_Rice_p1	H	7	pass
C5W6FACXX	5	GATCAGAA	TET_	2700100	Biofuel_Rice_p1	H	8	pass
C5W6FACXX	5	TGCATAGA	AC5_BB1_4	2484526	Biofuel_Rice_p1	H	9	pass
C5W6FACXX	5	GAACGAAT	CL8_LT2	2131348	Biofuel_Rice_p1	H	10	pass
C5W6FACXX	5	TTGGCGGA	NH_N46	2544876	Biofuel_Rice_p1	H	11	pass
C5W6FACXX	5	CGCCGCAT	BLANK	5417	Biofuel_Rice_p1	H	12	pass
C5W6FACXX	6	AGGC	BT7_IR64	2418859	Biofuel_Rice_p2	A	1	pass
C5W6FACXX	6	GATT	HDT7	2308946	Biofuel_Rice_p2	A	2	pass
C5W6FACXX	6	ACCGT	nep_meo_luong	2447545	Biofuel_Rice_p2	A	3	pass
C5W6FACXX	6	CGTCA	10L155	3160163	Biofuel_Rice_p2	A	4	pass
C5W6FACXX	6	TCGCA	09L28	3395093	Biofuel_Rice_p2	A	5	pass
C5W6FACXX	6	CGCAT	HD1	1909920	Biofuel_Rice_p2	A	6	pass
C5W6FACXX	6	TCATAGT	BB5_7	2108643	Biofuel_Rice_p2	A	7	pass
C5W6FACXX	6	TTACGAT	Lua_nuong	1907651	Biofuel_Rice_p2	A	8	pass
C5W6FACXX	6	GGCTAGA	Nep_do_duoi_trau	3470570	Biofuel_Rice_p2	A	9	pass
C5W6FACXX	6	ACAATGGA	BB7	3338495	Biofuel_Rice_p2	A	10	pass
C5W6FACXX	6	ACAAGAGT	RLT4586_NSF94	2385502	Biofuel_Rice_p2	A	11	pass
C5W6FACXX	6	GAACATGA	RLT4595_NSF161	2474597	Biofuel_Rice_p2	A	12	pass
C5W6FACXX	6	AGCATT	BB5	2683797	Biofuel_Rice_p2	B	1	pass
C5W6FACXX	6	CTCCGA	HDt8	2600134	Biofuel_Rice_p2	B	2	pass
C5W6FACXX	6	TTGGCA	Lua_ngoi	2450716	Biofuel_Rice_p2	B	3	pass
C5W6FACXX	6	CCACGT	10L164	2080867	Biofuel_Rice_p2	B	4	pass
C5W6FACXX	6	GATGTC	07L21	1881657	Biofuel_Rice_p2	B	5	pass
C5W6FACXX	6	TGTTAC	HDT2	1722741	Biofuel_Rice_p2	B	6	pass
C5W6FACXX	6	CAGTTA	Lua_nuong_2	2448869	Biofuel_Rice_p2	B	7	pass
C5W6FACXX	6	GCCTAT	Te_ka_cham_pi	2173614	Biofuel_Rice_p2	B	8	pass
C5W6FACXX	6	AGTGGC	Te_64	1369891	Biofuel_Rice_p2	B	9	pass
C5W6FACXX	6	TGACCT	BB3_7	2080788	Biofuel_Rice_p2	B	10	pass

Flowcell	lane	Barcode	ID	good barcoded reads	plate	row	col	Fail?
C5W6FACXX	6	TTGCAC	RLT4587_NSF98	1736789	Biofuel_Rice_p2	B	11	pass
C5W6FACXX	6	CTAGCT	RLT4596_NSF163	1691433	Biofuel_Rice_p2	B	12	pass
C5W6FACXX	6	AATCGTT	HDT5	2366738	Biofuel_Rice_p2	C	1	pass
C5W6FACXX	6	CTATGGA	BT7	2338356	Biofuel_Rice_p2	C	2	pass
C5W6FACXX	6	TACGGTA	Te_nuong	2503856	Biofuel_Rice_p2	C	3	pass
C5W6FACXX	6	ACTATGT	09L33	1642626	Biofuel_Rice_p2	C	4	pass
C5W6FACXX	6	CGTGAAT	11L108	2092663	Biofuel_Rice_p2	C	5	pass
C5W6FACXX	6	TTGCAGA	HDT7	2449038	Biofuel_Rice_p2	C	6	pass
C5W6FACXX	6	AACTTGT	Te_Ka_cham_pi__1	2136701	Biofuel_Rice_p2	C	7	pass
C5W6FACXX	6	TGACGTA	Pet_muong_canh_vang	2372932	Biofuel_Rice_p2	C	8	pass
C5W6FACXX	6	GCTATAA	Belecsec	2550627	Biofuel_Rice_p2	C	9	pass
C5W6FACXX	6	ATCGTAT	BB4_10	1958587	Biofuel_Rice_p2	C	10	pass
C5W6FACXX	6	TACTGAT	RLT4588_NSF100	2124318	Biofuel_Rice_p2	C	11	pass
C5W6FACXX	6	CTTGAGA	RLT4597_NSF170	2275387	Biofuel_Rice_p2	C	12	pass
C5W6FACXX	6	TCAAGTT	Huong_com	2335316	Biofuel_Rice_p2	D	1	pass
C5W6FACXX	6	GATCATA	OM5626	2350325	Biofuel_Rice_p2	D	2	pass
C5W6FACXX	6	GCATTGA	Khau_dien_lu	2589727	Biofuel_Rice_p2	D	3	pass
C5W6FACXX	6	CAGGTAT	09L140	1397465	Biofuel_Rice_p2	D	4	pass
C5W6FACXX	6	TGCAATA	11L12	2354789	Biofuel_Rice_p2	D	5	pass
C5W6FACXX	6	ATATCGT	BC15	1925031	Biofuel_Rice_p2	D	6	pass
C5W6FACXX	6	AGTCTAT	Tan_nhe_3	1893383	Biofuel_Rice_p2	D	7	pass
C5W6FACXX	6	GTCTGAA	Nep358	2679314	Biofuel_Rice_p2	D	8	pass
C5W6FACXX	6	ATCAGTT	Khau_lan_gan_mon	1881156	Biofuel_Rice_p2	D	9	pass
C5W6FACXX	6	CAGTTGA	1094_1	2355165	Biofuel_Rice_p2	D	10	pass
C5W6FACXX	6	TGTGCAA	RLT4589_NSF101	2214182	Biofuel_Rice_p2	D	11	pass
C5W6FACXX	6	CGACAGT	RLT4598_NSF171	1880179	Biofuel_Rice_p2	D	12	pass
C5W6FACXX	6	ACGTGTA	HT6__B5	3162222	Biofuel_Rice_p2	E	1	pass
C5W6FACXX	6	GATGCAT	Khau_giang	1811618	Biofuel_Rice_p2	E	2	pass
C5W6FACXX	6	CTAATGT	10L142	1941868	Biofuel_Rice_p2	E	3	pass
C5W6FACXX	6	GTCGATA	10L140	2285758	Biofuel_Rice_p2	E	4	pass
C5W6FACXX	6	TATACGT	11L17	1765397	Biofuel_Rice_p2	E	5	pass

Flowcell	lane	Barcode	ID	good barcoded reads	plate	row	col	Fail?
C5W6FACXX	6	GCGTAAT	P6_ST	1763079	Biofuel_Rice_p2	E	6	pass
C5W6FACXX	6	AGCGTTA	Tan_nhe_2	2640080	Biofuel_Rice_p2	E	7	pass
C5W6FACXX	6	ATCCGGA	T__th_m	2328467	Biofuel_Rice_p2	E	8	pass
C5W6FACXX	6	TCAGTAT	Khau_lan_gan_mon__1	2014814	Biofuel_Rice_p2	E	9	pass
C5W6FACXX	6	CAATGTT	PC6	1811522	Biofuel_Rice_p2	E	10	pass
C5W6FACXX	6	GTTACGA	RLT4590_NSF114	2519105	Biofuel_Rice_p2	E	11	pass
C5W6FACXX	6	TGCATAT	RLT4599_NSF182	1693891	Biofuel_Rice_p2	E	12	pass
C5W6FACXX	6	CAAGAAGT	P376	3214568	Biofuel_Rice_p2	F	1	pass
C5W6FACXX	6	GTCATGGT	OM3536	2461706	Biofuel_Rice_p2	F	2	pass
C5W6FACXX	6	AACAGTGA	10L182	2589270	Biofuel_Rice_p2	F	3	pass
C5W6FACXX	6	GTGCAAGA	10L144	2687896	Biofuel_Rice_p2	F	4	pass
C5W6FACXX	6	CAATAGGA	09L8	2365625	Biofuel_Rice_p2	F	5	pass
C5W6FACXX	6	TGCAGTGT	Te_meo	1882044	Biofuel_Rice_p2	F	6	pass
C5W6FACXX	6	AGGCTAGA	Pe_ngung__1	2552436	Biofuel_Rice_p2	F	7	pass
C5W6FACXX	6	CTAGTGGT	BB4_7	1885755	Biofuel_Rice_p2	F	8	pass
C5W6FACXX	6	GCTAGTGT	BB1_5	1962307	Biofuel_Rice_p2	F	9	pass
C5W6FACXX	6	AGTTGGCA	Blank	2857	Biofuel_Rice_p2	F	10	pass
C5W6FACXX	6	TCGCAAGT	RLT4591_NSF140	2166108	Biofuel_Rice_p2	F	11	pass
C5W6FACXX	6	CGATGTGT	RLT4600_NSF198	1843615	Biofuel_Rice_p2	F	12	pass
C5W6FACXX	6	AACGTAGA	AG_504	3284832	Biofuel_Rice_p2	G	1	pass
C5W6FACXX	6	CTCACGGA	Toc_lun	2243658	Biofuel_Rice_p2	G	2	pass
C5W6FACXX	6	TAGCGTGT	09L17	1791978	Biofuel_Rice_p2	G	3	pass
C5W6FACXX	6	ACGTAAGA	IRBB13	2312877	Biofuel_Rice_p2	G	4	pass
C5W6FACXX	6	CGTATGGT	10L150	2002653	Biofuel_Rice_p2	G	5	pass
C5W6FACXX	6	GTACGTGT	BB5	1711678	Biofuel_Rice_p2	G	6	pass
C5W6FACXX	6	TTCGAAGA	BB1_4	2419877	Biofuel_Rice_p2	G	7	pass
C5W6FACXX	6	AATACGGA	BB21	2401373	Biofuel_Rice_p2	G	8	pass
C5W6FACXX	6	TGACTGGT	Te_ruong__2	2293893	Biofuel_Rice_p2	G	9	pass
C5W6FACXX	6	GCGGATGT	RLT4584_NSF71	1908026	Biofuel_Rice_p2	G	10	pass
C5W6FACXX	6	CATTGAGA	RLT4592_NSF147	2358870	Biofuel_Rice_p2	G	11	pass
C5W6FACXX	6	GTAACAGA	RLT4601_NSF208	2536235	Biofuel_Rice_p2	G	12	pass
C5W6FACXX	6	AGCTTGGT	KG_4900	3265816	Biofuel_Rice_p2	H	1	pass

Flowcell	lane	Barcode	ID	good barcoded reads	plate	row	col	Fail?
C5W6FACXX	6	ACAGATGA	Om6377	3306328	Biofuel_Rice_p2	H	2	pass
C5W6FACXX	6	CAGTTGGT	09L15	2151687	Biofuel_Rice_p2	H	3	pass
C5W6FACXX	6	TGCAAGAA	11L162	3022906	Biofuel_Rice_p2	H	4	pass
C5W6FACXX	6	ACTCGAGA	AC5_CH133	3483678	Biofuel_Rice_p2	H	5	pass
C5W6FACXX	6	GGAGCTGT	BB4_11	2308690	Biofuel_Rice_p2	H	6	pass
C5W6FACXX	6	CTGAGTGT	Tan_nhe__1	1751915	Biofuel_Rice_p2	H	7	pass
C5W6FACXX	6	GATCAGAA	Pet_muong_canh_vang_1	3389749	Biofuel_Rice_p2	H	8	pass
C5W6FACXX	6	TGCATAGA	Nep_puoc_chia	3103103	Biofuel_Rice_p2	H	9	pass
C5W6FACXX	6	GAACGAAT	RLT4585_NSF74	2303430	Biofuel_Rice_p2	H	10	pass
C5W6FACXX	6	TTGGCGGA	RLT4594_NSF157	2526841	Biofuel_Rice_p2	H	11	pass
C5W6FACXX	6	CGCCGCAT	RLT4602_NSF385	1904002	Biofuel_Rice_p2	H	12	pass

Genome Info

original file: /workdir/genomes/public/rice/all.con
downloaded from
ftp://ftp.plantbiology.msu.edu/pub/data/Eukaryotic_Projects/o_sativa/annotation_dbs/pseudomolecules/version_7.0/all.dir/ on 19 September 2013.
md5sum:003aa04040ec2fa51cd8fde02a2515d4

chromosomes were renamed in the following manner for compatibility with the GBS pipeline:
removed leading "Chr", changed "ChrUn" to 100, changed "ChrSy" to 200

using the following commands:
sed 's/Chr//g' all.con | sed 's/Un/100/g' | sed 's/Sy/200/g' > all.renamed.con

resulting in the file:
/workdir/genomes/public/rice/all.renamed.con
of total size (bp) 381956586
with md5sum: d0218389927cbfec7908e53164fe8918

This file was indexed for use with bwa version 0.7.8-r455

BWA Alignment parameters

alignment generated with bwa Version: 0.7.8-r455

bwa aln -t 8 /workdir/genomes/public/rice/all.renamed.con
internal/02_mergedtagcounts/biofuel_rice_mccouch.fq >
internal/03_alignment/biofuel_rice_mccouch.sai

bwa samse /workdir/genomes/public/rice/all.renamed.con
internal/03_alignment/biofuel_rice_mccouch.sai
internal/02_mergedtagcounts/biofuel_rice_mccouch.fq >
internal/03_alignment/biofuel_rice_mccouch.sam

Alignment Results:

Total 4609509 tags
- 3649813 (79.2%) were aligned to unique positions
- 390089 (8.5%) were aligned to multiple positions
- 569607 (12.4%) could not be aligned

Resulting SNPs

- HapMap SNPs (unfiltered) : 639891
- HapMap SNPs (filtered) : 328915
- VCF SNPs: 1631277

VCFtools version [v0.1.11] was used to calculate Depth and Missingness from the unfiltered VCF file, taxa merged if available

	mean	median	standard_deviation
individual depth	7.817	7.262	2.798
site depth	7.817	6.503	6.699
individual missingness	0.179	0.172	0.093
site missingness	0.179	0.034	0.263

Multi Dimension Scaling (MDS) of genome-wide SNPs from the reference genome pipeline:

VCFtools version [v0.1.11] and Plink version [v1.07] were used to generate an MDS plot

- Genotypes were filtered to those with genotype quality 98 or higher (high confidence SNP calls) with VCFtools
- Biallelic SNPs were converted to PLINK format
- The remaining individuals/sites were filtered on missingness and allele frequency (with the same parameters as those used for the GBSHapMapFiltersPlugin
- Individuals removed from the analysis because of too much missing data:
    - BLANK:C5W6FACXX:5:250421518
    - Blank:C5W6FACXX:6:250421627
- Number of remaining sites for analysis: [230448] filtered, biallelic SNPs

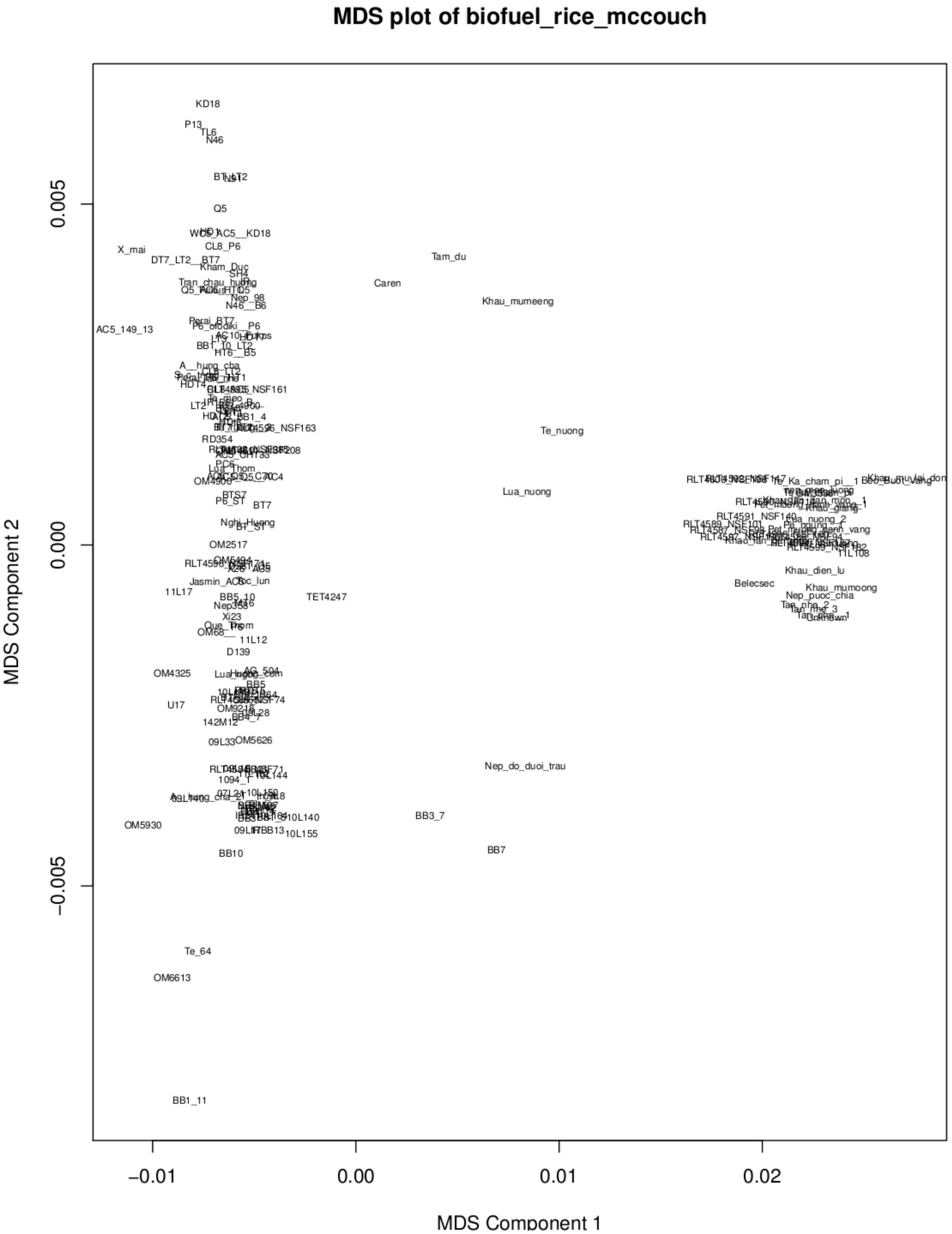
MDS plot of biofuel_rice_mccouch
MDS Component 2
MDS Component 1
0.005
0.000
−0.005
−0.01
0.00
0.01
0.02
KD18
P13
TL6
N46
Q5
X_mai
AC5_149_13
CL8_P6
Tam_du
Caren
Khau_mumeeng
Te_nuong
Lua_nuong
RD354
OM2517
11L17
TET4247
Belecsec
Khau_dien_lu
Khau_mumoong
Nep_puoc_chia
D139
OM4325
U17
142M12
09L33
OM5626
Nep_do_duoi_trau
BB3_7
OM5930
10L155
BB10
BB7
Te_64
OM6613
BB1_11

Genotyping by Sequencing Resources

GBS Overview

http://www.biotech.cornell.edu/brc/genomic-diversity-facility/services

GBS Frequently Asked Questions

http://www.biotech.cornell.edu/brc/genomic-diversity-facility/resources/faq

GBS Bioinformatics

http://www.biotech.cornell.edu/brc/genomic-diversity-facility/services/gbs-data-analysis

Training

http://www.biotech.cornell.edu/brc/genomic-diversity-facility/seminars-workshops-and-training

TASSEL

TASSEL 5.0

http://www.maizegenetics.net/gbs-overview#!tassel/c17q9

TASSEL 3.0/4.0

http://tassel.bitbucket.org/TasselArchived.html

Tassel Users Group

https://groups.google.com/forum/?fromgroups#!forum/tassel

Bibliography:

Elshire RJ, Glaubitz JC, Sun Q, Poland JA, Kawamoto K, et al. (2011) A Robust, Simple Genotyping-by-Sequencing (GBS) Approach for High Diversity Species. PLoS ONE 6(5): e19379. doi:10.1371/journal.pone.0019379

Glaubitz JC, Casstevens TM, Lu F, Harriman J, Elshire RJ, Sun Q, Bucker ES. (2013) TASSEL-GBS: A High Capacity Genotyping by Sequencing Analysis Pipeline. PLoS ONE 9(2): e90346 doi: 10.1371/journal.pone.0090346

Bradbury, P. J., Z. Zhang, D. E. Kroon, T. M. Casstevens, Y. Ramdoss, and E. S. Buckler. “TASSEL: Software for Association Mapping of Complex Traits in Diverse Samples.” Bioinformatics 23, no. 19 (June 22, 2007): 2633–2635.

Etter P, Bassham S, Hohenlohe PA, Johnson EA, Cresko W (2011). SNP Discovery and Genotyping for Evolutionary Genetics Using RAD Sequencing. Methods Mol Biol. 772:157-178. Doi: 10.1007/978-1-61779-228-1 9

Petr Danecek, Adam Auton, Goncalo Abecasis, Cornelis A. Albers, Eric Banks, Mark A. DePristo, Robert Handsaker, Gerton Lunter, Gabor Marth, Stephen T. Sherry, Gilean McVean, Richard Durbin and 1000 Genomes Project Analysis Group. The Variant Call Format and VCFtools. Bioinformatics, 2011

Purcell S, Neale B, Todd-Brown K, Thomas L, Ferreira MAR, Bender D, Maller J, Sklar P, de Bakker PIW, Daly MJ & Sham PC (2007) PLINK: a toolset for whole-genome association and population-based linkage analysis. American Journal of Human Genetics, 81, URL:http://pngy.mgh.harvard.edu/purcell/plink

R Development Core Team (2008). R: A language and environment for statistical computing. R Foundation for Statistical Computing, Vienna, Austria. ISBN 3-900051-07-0, URL http://www.R-project.org.
